# COVID-19 Pandemic Experiences and Symptoms of Pandemic-Associated Traumatic Stress Among Mothers in the US

**DOI:** 10.1001/jamanetworkopen.2022.47330

**Published:** 2022-12-16

**Authors:** Theresa M. Bastain, Emily A. Knapp, Andrew Law, Molly Algermissen, Lyndsay A. Avalos, Zoe Birnhak, Courtney Blackwell, Carrie V. Breton, Cristiane Duarte, Jean Frazier, Jody Ganiban, Paige Greenwood, Julie Herbstman, Ixel Hernandez-Castro, Julie Hofheimer, Margaret R. Karagas, Johnnye Lewis, David Pagliaccio, Bruce Ramphal, Darby Saxbe, Rebecca Schmidt, Carmen Velez-Vega, Xiaodan Tang, Ghassan B. Hamra, Amy Margolis, For the Environmental Influences on Child Health Outcomes Program Collaborators

**Affiliations:** 1 Department of Population and Public Health Sciences, University of Southern California Keck School of Medicine, Los Angeles; 2 Johns Hopkins Bloomberg School of Public Health, Baltimore, Maryland; 3 Columbia University Irving Medical Center, New York, New York; 4 Division of Research, Kaiser Permanente Northern California, Oakland, California; 5 Northwestern University Feinberg School of Medicine, Chicago, Illinois; 6 Columbia University–New York State Psychiatric Institute, New York; 7 University of Massachusetts Chan Medical School, Worcester; 8 Department of Psychological and Brain Sciences, Columbian College of Arts and Sciences, George Washington University, Washington, DC; 9 Columbia Mailman School of Public Health, New York City, New York; 10 Division of Neonatal-Perinatal Medicine, Department of Pediatrics, University of North Carolina at Chapel Hill, Chapel Hill; 11 Geisel School of Medicine at Dartmouth, Hanover, New Hampshire; 12 College of Pharmacy, University of New Mexico, Albuquerque; 13 Harvard University Medical School, New York, New York; 14 Dornsife College, University of Southern California, Los Angeles; 15 Department of Public Health Sciences, University of California, Davis, Davis; 16 Graduate School of Public Health, University of Puerto Rico Medical Sciences Campus, San Juan

## Abstract

**Importance:**

The primary outcomes of the COVID-19 pandemic on the mental health of women with children remain largely unknown.

**Objectives:**

To identify and describe clusters of mothers of children participating in the Environmental influences on Child Health Outcomes (ECHO) Program that characterize pandemic-associated hardships, coping mechanisms, and behaviors, and to evaluate associations between pandemic-associated hardships, coping strategies, and behavior changes with pandemic-associated traumatic stress symptoms.

**Design, Setting, and Participants:**

This multicenter cohort study investigated experiences during the COVID-19 pandemic between April 2020 and August 2021 among maternal caregivers of children participating in the ECHO Program. Data from self-identified mothers of ECHO-enrolled children from 62 US cohorts were included in analyses. Data were analyzed from November 2021 to July 2022.

**Exposures:**

The primary exposures were pandemic-associated changes in mothers’ health, health care utilization, work and finances, coping strategies, and health-associated behaviors. Exposures were assessed via a self-reported questionnaire designed by ECHO investigators.

**Main Outcomes and Measures:**

The primary outcome was the total symptoms score of pandemic-associated traumatic stress (PTS), defined as the number of items endorsed at least sometimes or more frequently, from a 10-item self-report measure.

**Results:**

The study surveyed 11 473 mothers (mean [SD] age, 37.8 [7.4] years; 342 American Indian [2.98%], 378 Asian [3.29%], 1701 Black [14.83%], and 7195 White [62.71%]; 2184 with Hispanic/Latina ethnicity [19.04%]) and identified 2 clusters that best characterized their COVID-19 pandemic experiences—one characterized by higher life disruptions (eg, to work and health care), higher social isolation, more coping behaviors to mitigate the outcomes of the pandemic, and more changes to their health behavior routines (high change [1031 mothers]) and the other characterized by lower changes (low change [3061 mothers]). The high change cluster was more socioeconomically advantaged and reported higher PTS (mean [SD] number of symptoms, 3.72 [2.44] vs 2.51 [2.47]). Across both clusters, higher pandemic-associated hardships, coping mechanisms, and behavior changes were associated with higher PTS, and these associations were greater in the low change cluster.

**Conclusions and Relevance:**

In this study of more than 11 000 US mothers, associations between socioeconomic factors, stressful life events, and mental health sequelae were complex. Accordingly, programs, policies, and practices targeting mental health during public health crises such as the COVID-19 pandemic should consider the range and configuration of hardships in designing the most effective interventions to mitigate long-term outcomes.

## Key Points

**Question:**

Is there an association between greater pandemic-associated hardships, coping strategies, and health behavior changes with pandemic-associated traumatic stress symptoms among mothers in the US?

**Findings:**

This cohort study including 11 473 participants found that higher pandemic-associated hardships, coping mechanisms, and behavior changes were associated with greater significant symptoms of pandemic-associated traumatic stress in mothers from socioeconomically, ethnically, and racially diverse backgrounds.

**Meaning:**

These findings suggest that the complex associations between sociodemographic factors, stressful life events, and mental health sequelae should be considered in future studies examining the long-term outcomes of the COVID-19 pandemic and other traumatic life events.

## Teaser

This cohort study evaluates the association between COVID-19 pandemic experiences and symptoms of pandemic-associated traumatic stress among mothers in the US.

## Introduction

Mothers may be particularly susceptible to psychological stress effects from the COVID-19 pandemic.^1,2,3^ School closures impact working mothers, leading to loss of income and complimentary meals in school settings, unexpected childcare expenses, and gaps in adequate technology and quiet space for remote learning.^4,5,6,7,8,9,10,11,12,13^ One study^14^ found that mothers with a child younger than 18 years were among the most likely to report that the COVID-19 pandemic has had an adverse impact on their mental health. It is critical to understand the associations between pandemic hardships and mental health of US mothers.

Studies^15,16,17,18^ have consistently documented the disproportionate impacts of COVID-19 morbidity and mortality on US communities of color. Members of minoritized groups and individuals from communities excluded from economic opportunities may also be at increased risk for adverse mental health impacts from the pandemic.^14,19,20,21,22,23,24^ However, gaps remain in understanding how multiple pandemic-associated hardships, health behavior changes, and coping strategies may co-occur by levels of socioeconomic advantage and/or racial and ethnic group and how these factors are associated with traumatic stress symptoms. The Environmental influences on Child Health Outcomes (ECHO) Program^25,26^ follows a diverse multicohort sample and provides an opportunity to understand associations between the COVID-19 pandemic and mothers’ psychological health. The objectives of the current analysis were to (1) characterize clusters of mothers according to pandemic-associated hardships, coping strategies, and behavior changes; (2) describe sociodemographic characteristics of the clusters; and (3) evaluate the association between these pandemic experiences and pandemic-associated traumatic stress (PTS) overall and within clusters. We hypothesized that we would identify 2 or more clusters of mothers with similar pandemic experiences that vary by sociodemographic characteristics such as income, education, and race and ethnicity. We further hypothesized that mothers with greater pandemic hardships, coping strategies, and health behavior changes would report higher PTS.

## Methods

**Figure 1.**
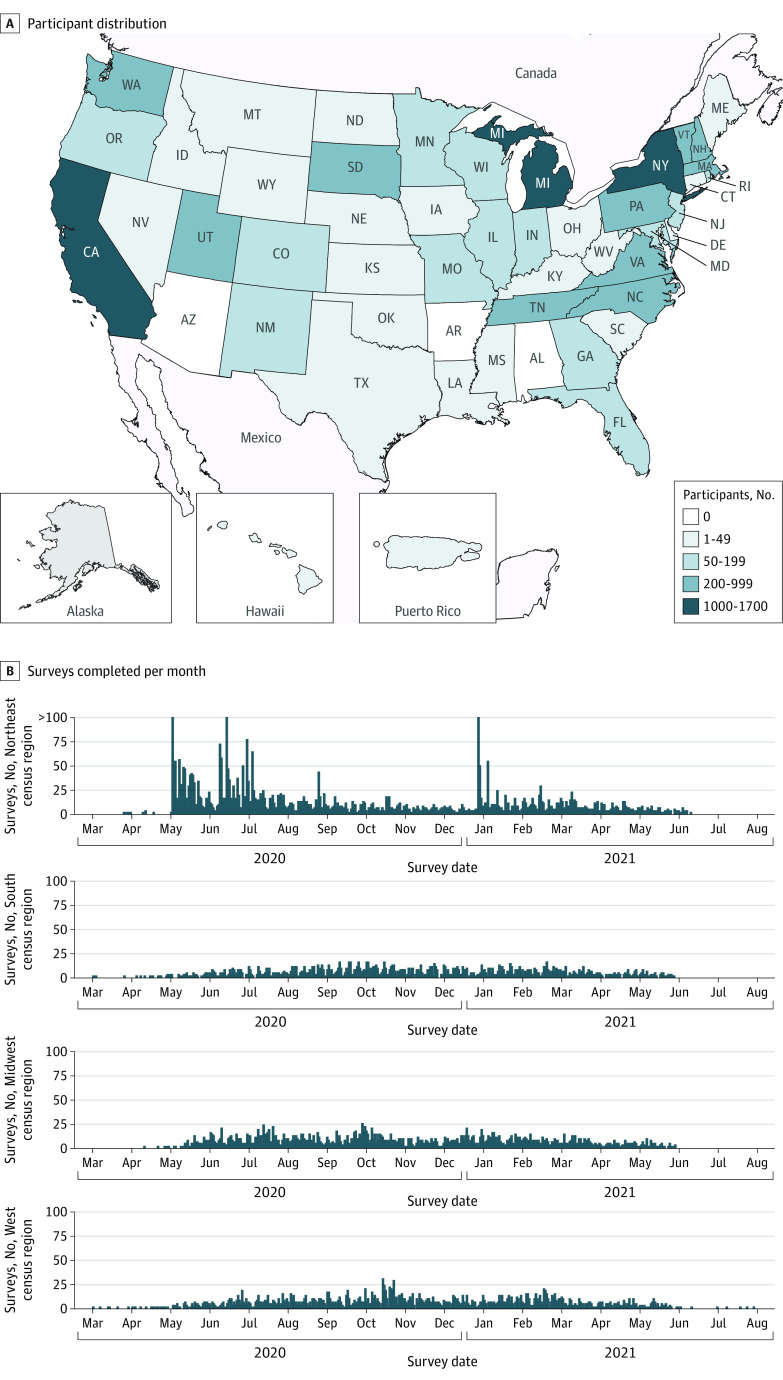
Participant Residential Locations and Timing of COVID-19 Survey by US Census Regions Panel A shows participants representing 62 Environmental influences on Child Health Outcomes cohorts and residing in 46 US states and Puerto Rico at the time of survey administration. Panel B shows participants who completed COVID-19 questionnaires across a 16-month period from March 2020 through August 2021. The figure displays the number of surveys completed per month across each of 4 US Census regions.

ECHO is a consortium of 69 longitudinal birth and pediatric cohort studies focusing on 5 broad child health outcomes (eTable 1 in Supplement 1).^26,27^ Pregnant individuals and children from across the US were enrolled into ECHO (Figure 1). Participants provided written informed consent for individual cohort and ECHO Program enrollment. Central (Western Institutional Review Board) and/or cohort-specific institutional review boards oversee ECHO’s human participants research. This study followed the Strengthening the Reporting of Observational Studies in Epidemiology (STROBE) reporting guidelines for cohort studies.

### Study Population

We included caregivers of ECHO child participants who indicated “mother” on the ECHO COVID-19 adult questionnaire between May 2020 and August 2021 (eFigure 1 in Supplement 1). The earliest date of administration was selected when mothers completed more than 1 COVID-19 questionnaire (21% participants completed more than one survey).

### Measures

#### COVID-19 Questionnaire

In March and April 2020, ECHO developed and released a COVID-19 questionnaire to assess pandemic experiences including families’ health, health care utilization, parental work and finances, health-associated behaviors, and coping strategies.^28^ A total of 13 pandemic experiences were examined (mother or household member testing positive for COVID-19 [2 items]; changes to health care [1 item]; increased social isolation [5 items]; and changes to the mother’s and/or partner’s work [5 items]). A total of 11 coping strategies and 7 behavior and /or lifestyle changes were included. See the eAppendix in Supplement 1 for a copy of the survey.

The ECHO COVID-19 questionnaire also includes the 10-item Pandemic-associated Traumatic Stress Scale (PTSS), a novel measure of traumatic stress symptoms specific to the COVID-19 pandemic^29^ based on the *Diagnostic and Statistical Manual of Mental Disorders* (Fifth Edition)^30^ criteria for acute stress disorder. Acute stress disorder is characterized by the presence of symptoms of traumatic stress following exposure to an event that threatens one’s life or the life of a loved one. Items comprise 5 domains of acute stress reactions: intrusion, negative mood, disassociation, avoidance, and difficulty regulating arousal. Responses to each item range from not at all (1) to very often (5). The primary outcome of this study was the total symptoms score to signify frequently occurring symptoms, in which each item was rescored as 1 if participants sometimes, often, or very often reported the symptom or 0 if a symptom was rarely or not at all reported and then these were summed (range, 0-10). We also included 2 secondary outcomes (eTable 2, eTable 3, and eTable 4 in Supplement 1): (1) a total sum score (range, 5-50) that reflects general stress severity, and (2) a total symptom categories score that reflects the number of symptom categories endorsed at a clinically significant level (range, 0-5).

#### Sociodemographic Factors and Geographic Location

Education, household income within 5 years of survey administration, and cohabitation status were collected by self-report. Maternal race and ethnicity were collected by self-report using predefined categories (American Indian, Asian, Black, multiple race, other race [which includes Native Hawaiian/Pacific Islander and other race], and White) and were included in analyses as a proxy for exposure to structural racism, which places members of racial and ethnic minority groups at greater risk of pandemic-associated hardships. To adjust for regional influences on the pandemic (eg, differences in mitigation policies and infection rates), we included state of residence within 5 years of questionnaire date; participants who did not report a residential address within this time frame were assigned the state of their cohort recruitment site.

### Statistical Analysis

Additional detail is provided in eMethods in Supplement 1. Briefly, we characterized clusters of mothers according to their responses to 31 pandemic experiences. We used k-means clustering,^31,32^ an unsupervised machine learning method that groups individuals according to similarities in patterns of responses to specified inputs. The optimal number of clusters was selected according to a Silhouette analysis.^33,34^ We then described the sociodemographic characteristics of these clusters.

We conducted sparse partial least squares (SPLS) regression^35^ analysis of the cross-sectional association between COVID-19 pandemic experiences with PTS, adjusting for cohort recruitment site and state of residence. SPLS is a supervised machine learning approach that conducts variable selection and shrinkage according to whether inputs are determined to be associated with the outcome, relative to the other inputs in the model. Variables not retained in the model are described as dropped in results tables. Bootstrapping was used to calculate 95% CIs. We determined whether the association between responses to pandemic experiences and PTS differed by cluster membership by running stratified, cluster-specific SPLS models.

Estimates of association from SPLS models are interpreted as the change in the number of PTS symptoms among those responding yes to a COVID hardship or coping strategy compared with those responding no. We discuss differences between clusters if (1) hardship is retained in only 1 cluster and its 95% CI does not include 0.00 or (2) if the 95% CI for the quantified difference in the estimates between clusters does not include 0.00; the methods to calculate the latter are described in the eMethods in Supplement 1.^36^ Finally, all data and statistical code necessary to replicate the results presented are maintained by and available from the ECHO Data Analysis Center. Data were analyzed in R, version 3.6.2 (R Project for Statistical Computing) from November 2021 to July 2022.

## Results

COVID-19 questionnaires were completed by 14 419 adult caregivers. Participants were excluded if they indicated “biological father,” “participant,” or “other” respondent (15 participants) or if they were pregnant with the ECHO child participant (495 participants). The final study sample included 11 473 mothers of ECHO children from 62 cohorts. An additional 675 participants were missing all items in the PTSS, and 123 were missing state of residence and therefore were excluded from the SPLS regression (eFigure 1 in Supplement 1).

Among 11 473 mothers of ECHO children from 60 cohorts included in the cluster analysis, 342 (2.98%) were American Indian, 378 (3.29%) Asian, 1701 (14.83%) Black, and 7195 (62.71%) were white race; 2184 (19.04%) mothers reported Hispanic ethnicity (Table 1). Most mothers attended at least some college (8788 participants [76.60%]), and 1174 participants (10.23%) reported a household income of less than $30 000 per year. The mean (SD) total significant symptoms was 3.40 (2.51). The mean (SD) total sum of PTS was 21.02 (6.95), and the mean (SD) total symptom categories score was 2.06 (1.44) (eTable 2 in Supplement 1).

**Table 1.** Sociodemographic Characteristics of Mothers of Environmental influences on Child Health Outcomes Children, Overall and By Cluster

Demographic characteristics	Participants, No. (%)		
	Overall (N = 11 473)	Low change Cluster (n = 3061)	High change Cluster (n = 8412)
Maternal race			
American Indian	342 (2.98)	162 (5.29)	180 (2.14)
Asian	378 (3.29)	55 (1.80)	323 (3.84)
Black	1701 (14.83)	709 (23.16)	992 (11.79)
Multiple race	1008 (8.79)	209 (6.83)	799 (9.50)
Other race^a^	279 (2.43)	52 (1.70)	227 (2.70)
White	7195 (62.71)	1656 (54.10)	5539 (65.85)
Missing	570 (4.97)	218 (7.12)	352 (4.18)
Maternal ethnicity			
Not Hispanic	9086 (79.19)	2459 (80.33)	6627 (78.78)
Hispanic	2184 (19.04)	543 (17.74)	1641 (19.51)
Lifetime highest attained maternal education			
Some college and above	8788 (76.60)	1946 (63.57)	6842 (81.34)
High School, GED, equivalent, or less than high school	1890 (16.47)	859 (28.06)	1031 (12.26)
Missing	795 (6.93)	256 (8.36)	539 (6.41)
Annual household income, $			
<30 000	1174 (10.23)	566 (18.49)	608 (7.23)
30 000-49 999	830 (7.23)	317 (10.36)	513 (6.1)
50 000-74 999	1012 (8.82)	317 (10.36)	695 (8.26)
75 000-99 999	899 (7.84)	223 (7.29)	676 (8.04)
≥100 000	3181 (27.73)	418 (13.66)	2763 (32.85)
Missing	4377 (38.15)	1220 (39.86)	3157 (37.53)
Maternal marital status			
Married or living with partner	5929 (51.68)	1410 (46.06)	4519 (53.72)
Single	2008 (17.50)	831 (27.15)	1177 (13.99)
Missing	3536 (30.82)	820 (26.79)	2716 (32.29)
COVID-19 pandemic-associated financial concerns caused stress			
Yes	3933 (34.28)	925 (30.22)	3008 (35.76)
No	6769 (59.00)	2064 (67.43)	4705 (55.93)
Missing	771 (6.72)	72 (2.35)	699 (8.31)
US Census region			
Northeast	4296 (37.85)	955 (31.46)	3341 (40.19)
South	1864 (16.42)	603 (19.86)	1261 (15.17)
Midwest	2675 (23.57)	972 (32.02)	1703 (20.48)
West	2515 (22.16)	506 (16.67)	2009 (24.16)
Missing	123 (1.07)	25 (<1)	98 (1.17)
Total Symptoms Scores, mean (SD)^b^	3.40 (2.51)	2.51 (2.47)	3.72 (2.44)

^a^ Other race category includes Native Hawaiian/Pacific Islander (less than 1% of sample) and those respondents who selected the “other race” category (2%).

^b^ The COVID-19 Pandemic-Associated Traumatic Stress Scale (PTSS) is a 10-item measure of pandemic-associated symptoms. Total Symptoms score reflects the number of symptoms reported at a clinically relevant level (sometimes, often or very often; range 0 to 10) by the participant.

**Figure 2.**
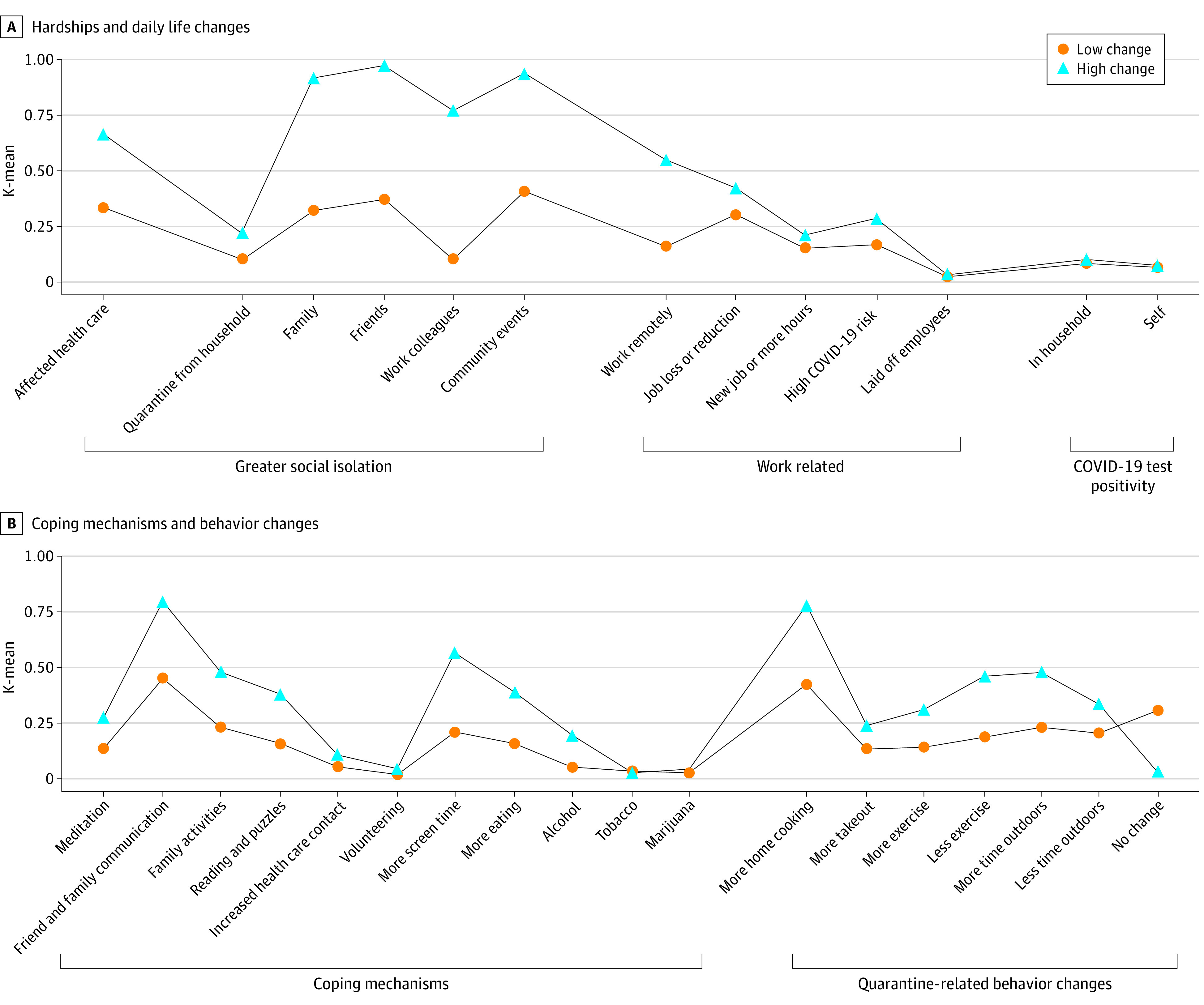
Clusters of Pandemic-Associated Hardships, Coping Strategies, and Behavior Changes in Mothers of Environmental influences on Child Health Outcomes Children Panel A shows hardships/daily life impacts. Panel B shows coping mechanisms and behavior changes.

We found that 2 clusters best fit COVID-19 pandemic experiences (Figure 2 and eFigure 2 in Supplement 1). There were differences between the 2 clusters with respect to social isolation, work-associated outcomes, coping mechanisms, and behavior changes (eTable 5 in Supplement). Timing of survey administration was similar between the 2 clusters.

### Descriptive Characteristics by Cluster Membership

Sociodemographic characteristics are shown in Table 1, for the overall study sample and by cluster membership. Cluster 1, labeled the “lower changes in health behaviors, fewer coping strategies, and lower social isolation” (low change) cluster, contained 3061 mothers and was characterized by lower educational attainment: 859 (28.06%) had a high school diploma or equivalent or lower level of education vs 1031 (12.26%) in Cluster 2, labeled the “higher changes in health behaviors, more coping strategies, and higher social isolation” (high change) cluster. The low change cluster also had lower incomes (566 [18.49%] with <$30 000 per year vs 608 [7.23%] in the high change cluster) and a higher proportion of single/noncohabitating mothers (831 participants [27.15%] vs 1177 participants [13.99%] in the high change cluster). The low change cluster had a higher proportion of Black mothers (709 participants [23.16%] vs 992 participants [11.79%]) and mothers from indigenous communities (162 participants [5.29%] vs 180 participants [2.14%]).

The high change cluster contained 8412 mothers and was characterized by higher incomes, higher education, and higher cohabitation. The high change cluster was more likely to report financial concerns as a source of stress (3008 participants [35.76%] vs 925 participants [30.22%]) and had a higher proportion of White mothers (5539 participants [65.85%] vs 1656 participants [54.10%]). Both clusters had similar distributions of Latina and Hispanic participants.

Mothers in the high change cluster were more likely to report that the pandemic affected their health care and to report greater social isolation (Figure 2). Mothers in the high change cluster also were more likely to report the use of certain coping mechanisms. Both clusters reported similar rates of COVID-19 infection in the household and similar work changes.

The mean (SD) number of total symptoms of PTS was higher among mothers in the high change than the low change cluster (3.72 [2.44] vs 2.51 [2.47]) (Table 1). eFigure 3 in Supplement 1 shows the number of total symptoms of PTS stratified by cluster. Both clusters contained mothers who frequently endorsed all 10 symptoms; however, the shape of distributions of total symptoms of PTS differed by cluster. The distribution of PTS symptoms among the low change cluster was right-skewed, whereas the distribution was more normal in the high change cluster.

### Associations of PTS With Pandemic Experiences

Table 2 presents the results of associations of pandemic exposures with total significant (ie, frequent) symptoms of PTS. Overall, several pandemic hardships were associated with increased PTS, with social isolation, and COVID-19 impacting one’s health care having the largest positive associations. In some cases, associations with hardships differed by cluster. Among the low change cluster, COVID-19 infection was associated with higher PTS. Mothers who reported that the COVID-19 pandemic affected their health care, job loss, or reduction of hours showed higher PTS in both groups, but associations were greater within the low change cluster (eg, for COVID–affected health care, β = 0.42 [95% CI, 0.32 to 0.54] for low change and β = 0.26 [95% CI, 0.21 to 0.32] for high change). Reporting less contact with friends was associated with lower PTS among the low change cluster (β = –0.11; 95% CI, –0.23 to–0.01). Social isolation from community events was not associated with PTS in either cluster.

**Table 2.** Associations Between Pandemic Hardships, Coping Strategies, and Behavior Changes and Total Symptoms Score of Pandemic-Associated Traumatic Stress by Cluster and Overall in Mothers of Environmental influences on Child Health Outcomes Children

Pandemic hardships, coping strategies, and behavior changes	Total Symptoms Score, β coefficient (95% CI)^a^^,^^b^			
	Overall (n = 10 675)	Low change Cluster (n = 2882)	High change Cluster (n = 7793)	Difference between clusters^c^
Hardships/daily life impacts				
COVID affected health care	0.33 (0.30 to 0.37)	0.42 (0.32 to 0.54)	0.26 (0.21 to 0.32)	0.16 (0.04 to 0.17)
Social isolation				
Quarantine from household	0.13 (0.10 to 0.16)	0.15 (0.07 to 0.23)	0.12 (0.06 to 0.16)	0.03 (–0.07 to 0.04)
Less contact with family	0.26 (0.24 to 0.29)	0.05 (–0.05 to 0.15)	0.07 (0.04 to 0.09)	0.02 (–0.09 to 0.13)
Less contact with friends	0.20 (0.18 to 0.23)	–0.11 (–0.23 to –0.01)	Dropped	NA
Less contact with colleagues	0.27 (0.23 to 0.30)	Dropped	0.05 (0.01 to 0.10)	NA
Fewer community events	0.18 (0.16 to 0.21)	Dropped	Dropped	NA
Work related				
Work remotely	0.10 (0.07 to 0.14)	Dropped	–0.07 (–0.12 to –0.02)	NA
Job loss or fewer hours	0.25 (0.21 to 0.28)	0.41 (0.31 to 0.54)	0.25 (0.20 to 0.31)	0.17 (0.04 to 0.29)
New job or fewer hours	Dropped	Dropped	Dropped	NA
High COVID risk	0.11 (0.08 to 0.14)	0.13 (0.04 to 0.22)	0.08 (0.03 to 0.14)	0.04 (–0.06 to 0.15)
Laying off employees	Dropped	Dropped	Dropped	NA
COVID infection				
COVID in household	0.05 (0.03 to 0.08)	0.14 (0.07 to 0.22)	Dropped	NA
Adult COVID positive	Dropped	0.10 (0.04 to 0.17)	Dropped	NA
Coping strategies				
Meditation	0.15 (0.12 to 0.18)	0.19 (0.11 to 0.29)	0.13 (0.08 to 0.18)	0.06 (–0.04 to 0.16)
Talking with friends or family	0.22 (0.19 to 0.25)	0.42 (0.31 to 0.53)	Dropped	NA
More family activities	Dropped	0.16 (0.07 to 0.25)	–0.25 (–0.30 to –0.20)	0.40 (0.30 to 0.50)
Increased time reading books or doing puzzles	0.05 (0.02 to 0.09)	0.09 (0.01 to 0.16)	–0.08 (–0.13 to –0.02)	0.17 (0.07 to 0.26)
Talking to health care practitioner	0.17 (0.14 to 0.19)	0.17 (0.11 to 0.24)	0.22 (0.18 to 0.26)	0.04 (–0.03 to 0.12)
Volunteering	Dropped	Dropped	Dropped	NA
Increased screen time	0.44 (0.40 to 0.47)	0.45 (0.35 to 0.55)	0.42 (0.37 to 0.49)	0.03 (–0.09 to 0.14)
Eating more often	0.41 (0.38 to 0.45)	0.43 (0.35 to 0.53)	0.46 (0.41 to 0.52)	0.03 (–0.08 to 0.14)
Drinking alcohol	0.22 (0.19 to 0.25)	0.16 (0.10 to 0.22)	0.26 (0.22 to 0.32)	0.10 (0.03 to 0.18)
Using tobacco	0.05 (0.04 to 0.06)	0.11 (0.06 to 0.16)	0.06 (0.04 to 0.08)	0.05, (0.00 to 0.10)
Using marijuana	0.07 (0.05 to 0.09)	0.07 (0.03 to 0.11)	0.09 (0.07 to 0.12)	0.03 (–0.02 to 0.08)
Behavior changes				
More home-cooked meals	0.16 (0.13 to 0.19)	0.22 (0.12 to 0.32)	Dropped	NA
More takeout food	0.13 (0.10 to 0.16)	0.17 (0.11 to 0.25)	0.11 (0.06 to 0.16)	0.07 (–0.02 to 0.15)
More physical exercise	Dropped	Dropped	–0.12 (–0.17 to –0.07)	NA
Less physical exercise	0.33 (0.30 to 0.37)	0.26 (0.16 to 0.35)	0.34 (0.30 to 0.39)	0.08 (–0.02 to 0.19)
More time outdoors	Dropped	Dropped	–0.17 (–0.21 to –0.12)	NA
Less time outdoors	0.28 (0.24 to 0.31)	0.27 (0.19 to 0.36)	0.32 (0.29 to 0.37)	0.06 (–0.04 to 0.16)
No changes in behavior	–0.19 (–0.21 to –0.16)	–0.41 (–0.49 to –0.32)	Dropped	NA

Abbreviation: NA, not applicable.

^a^ The COVID-19 Pandemic-Related Traumatic Stress Scale is a 10-item measure of pandemic-related symptoms. Total Symptoms score reflects the number of symptoms reported at a clinically relevant level by the participant (range 0 to 10).

^b^ Sparse partial least squares (SPLS) models conduct variable selection and shrinkage for variables that are determined to be relevant variables of the outcome, relative to the other inputs in the model. This results in some variables being dropped from the models, as noted in the table. Reporting a new job or more hours, laying off employees, and volunteering were dropped in the overall SPLS model as well as cluster specific models.

^c^ The difference between clusters calculation is described in the eMethods in Supplement 1. Differences cannot be calculated in situations where the variable is dropped from either cluster-specific model, which is noted in the table as NA.

Overall, PTS was positively associated with engagement in coping strategies. The coping mechanisms of more screen time, more eating, using marijuana or tobacco, meditating, and increased contact with a health care practitioner showed associations with PTS of similar magnitude across both clusters. Talking more with family and friends was associated with higher PTS in the low change cluster (β = 0.42 [95% CI, 0.31 to 0.53]). Drinking alcohol to cope with the pandemic was associated with higher PTS in both groups, but a larger association was observed among the high change cluster (β = 0.26 [95% CI, 0.22 to 0.32] vs β = 0.16 [95% CI, 0.10 to 0.22] in the low change cluster). Increased time reading books or doing puzzles and more family activities were associated with higher PTS among the low change cluster (β = 0.09 [95% CI, 0.01 to 0.16] and β = 0.16 [95% CI, 0.07 to 0.25], respectively) but lower PTS in the high change cluster (β = –0.08 [95% CI, –0.13 to –0.02] and β = –0.25 [95% CI, –0.30 to –0.20], respectively).

Changes in multiple health behaviors were associated with higher PTS across the full sample. We found that changes in health behaviors, including less exercise, less time outdoors, and eating more takeout food, showed similar associations with higher PTS in both clusters (ie, β = 0.32 [95% CI, 0.29 to 0.37] in high change and β = 0.27 95% CI, 0.19 to 0.36] in low change for spending less time outdoors). However, more physical exercise and more time outdoors were associated with lower PTS only in the high change cluster (β = −0.12 [95% CI, −0.17 to −0.07] and β = −0.17 [−0.21 to −0.12], respectively). Eating more home-cooked meals was associated with higher PTS only in the low change cluster (β = 0.22 [95% CI, 0.12 to 0.32]). Notably, reporting no behavior changes was associated with lower PTS only in the low change cluster (β = −0.41 [95% CI, −0.49 to −0.32]). Results for the secondary outcomes of total sum and total categories scores are presented in eTables 4 and 5 in Supplement 1 and are largely consistent with results for total symptoms.

## Discussion

In a large diverse nationwide sample of more than 11 000 mothers, this cohort study found 2 distinct patterns of pandemic experiences: 1 with greater changes and 1 with fewer changes compared with prepandemic. These 2 clusters of experiences varied according to levels of socioeconomic advantage. Mothers in the more advantaged high change cluster reported greater life disruptions, social isolation, and coping behaviors to mitigate the effects of the pandemic and changes to their health behavior routines compared with mothers in the less advantaged, low change cluster. Mothers in the high change cluster also reported more PTS. Across both clusters, we found that higher pandemic-associated hardships, coping mechanisms and behavior changes were associated with higher PTS. However, we observed important differences in associations of pandemic hardships and coping mechanisms with PTS between the 2 clusters.

Studies have shown that persons with higher socioeconomic advantage have had a different pandemic experience from those with lower socioeconomic advantage.^21^ The ability to work remotely and quarantine away from other household members is a privilege often not available to persons in lower income households or essential workers. However, along with the privilege of working remotely and being able to quarantine away from other household members, considerable disruption in day-to-day life was also experienced in the high change cluster including greater social isolation from family, friends, and colleagues, who are important sources of social support when confronted with a major traumatic life event. A surprising result was that mothers in the low change cluster reported higher PTS when they spent more time with family. However, spending greater time with family might be stressful for mothers with lower socioeconomic advantage, especially if they reside in smaller homes where they may be unable to quarantine away from family members.

Overall, we found that mothers who reported higher patterns of both adaptive and maladaptive coping mechanisms also reported higher PTS. Our results are partially consistent with a study investigating behavioral coping impacts on COVID-19-associated distress among 4412 pregnant and postpartum mothers from 9 US states.^37^ This study showed that mothers with high levels of passive coping strategies (eg, increased screen time) had elevated symptoms of distress, but women with high levels of active coping strategies (eg, greater social support), showed greater resiliency. Our results suggest that increased time engaging in quiet activities or in more family activities were associated with lower PTS in the high change cluster. Another study conducted in 2020 among 524 expectant and new mothers found that resilience coping factors including using more technology to maintain support networks, more exercise, and time spent outdoors were associated with lower severity of mental health symptoms.^38^ In our study, we showed that more physical exercise and more time spent outdoors were associated with lower PTS in the high change cluster. Importantly, these activities were associated with higher PTS in the low change cluster. In the low change cluster, higher PTS was associated with talking with friends or family and more home-cooked meals but lower PTS was associated with mothers reporting no health behavior changes. These results suggest that change itself may be a key factor associated with PTS, or, alternatively, that we may not have asked the right questions about what coping mechanisms are effective in communities with lower socioeconomic advantage.

### Strengths and Limitations

Our study has many unique strengths. To our knowledge, this is the largest study of diverse mothers that investigated pandemic experiences from nearly all 50 US states and Puerto Rico. The study also covered a broad temporal period of the pandemic from the early lockdown phase through the Delta variant phase. In addition, we used both unsupervised (clustering) and supervised (SPLS) statistical analytic approaches, which provided insight into similarities according to lived experiences and allowed us to focus on variables with the strongest demonstrated associations with the outcome.

There are also several important limitations to consider. We limited our sample to mothers as they were the largest caregiver respondent group; we were, therefore, unable to examine differences in PTS experienced by fathers or other caregivers. Although all caregivers of children experienced pandemic-associated changes, mothers have experienced well-documented childcare and job-associated impacts from the pandemic.^1,2,3,12,13^ Other limitations include the lack of data about prior psychiatric diagnoses that may influence PTS, the use of a novel PTS scale, and lack of pandemic experiences of other household members, as our questionnaires focus on the caregiver and ECHO child.

As this is a cross-sectional study nested in a prospective cohort study, were unable to address the temporality of associations between traumatic stress and pandemic experiences. Similarly, respondents concurrently reported PTS symptoms with pandemic experiences that may have occurred more than a year before reporting, which could introduce recall bias. We were unable to directly assess whether higher rates of regional COVID-19 infection or local health orders influenced participation as cohorts administered the questionnaire according to their own visit schedules. We examined the impact of analyzing the first or last survey among the approximately 20% of participants who completed more than 1, and found that our results were largely unchanged (data not shown). Therefore, we are confident that our wide temporal and geographic variation broadly captured the pandemic experience of US mothers with children.

## Conclusions

In this nationwide cohort study, we found that mothers who reported greater overall pandemic-associated disruptions also reported more PTS symptoms. Our results suggest that the complex relationships between sociodemographic factors, stressful life events, and mental health sequelae should be considered in future studies examining the long-term effects of the COVID-19 pandemic and other traumatic life events. Similarly, programs, policies and practices targeting mental health during public health crises, such as the COVID-19 pandemic, need to consider the range and configuration of hardships in designing the most effective interventions to mitigate long-term effects.

## Notes

Supplement 1. **eTable 1. **Description of ECHO Cohorts Included in Analysis **eFigure 1. **Flowchart of Study Participants and Survey Selection **eAppendix. **Survey **eTable 2. **Secondary Pandemic-Associated Traumatic Stress Outcomes in Mothers of ECHO Children by Cluster and Overall **eTable 3. **Association Between Pandemic-Associated Hardships/Daily Life Changes, Coping Strategies, and Behavior Changes and Pandemic-Associated Traumatic Stress Total Sum Score Among Mothers of ECHO Children **eTable 4. **Association Between Pandemic-Associated Stressors, Coping Strategies, and Behavior Changes and Pandemic-Associated Traumatic Stress Symptoms Categories
eMethods
**eFigure 2. **Optimal Number of Clusters by Total Within Sum of Squares (Panel A) and Silhouette Width (Panel B) **eTable 5. **Pandemic-Associated Hardships, Coping Strategies and Behavior Changes By Cluster and Overall Reported by ECHO Mothers **eFigure 3. **Number of Total Significant Symptoms of Pandemic-Associated Traumatic Stress by Cluster in Mothers of ECHO Children
eReferences

Supplement 2.
Nonauthor Collaborators

Supplement 3.
Data Sharing Statement

## References

[1] Zamarro G, Prados MJ. Gender differences in couples’ division of childcare, work and mental health during COVID-19. Rev Econ Househ. 2021;19(1):11-40. doi:10.1007/s11150-020-09534-7 33488316 PMC7811157

[2] Connor J, Madhavan S, Mokashi M, . Health risks and outcomes that disproportionately affect women during the COVID-19 pandemic: a review. Soc Sci Med. 2020;266:113364. doi:10.1016/j.socscimed.2020.113364 32950924 PMC7487147

[3] Lambregtse-van den Berg M, Quinlivan JA. COVID-19, global inequality, and mental health in childbearing women: how to mitigate the triple hit? J Psychosom Obstet Gynaecol. 2021;42(4):259-260. doi:10.1080/0167482X.2021.2001933 34809539

[4] Basurto-Dávila R, Garza R, Meltzer MI, . Household economic impact and attitudes toward school closures in two cities in Argentina during the 2009 influenza A (H1N1) pandemic. Influenza Other Respir Viruses. 2013;7(6):1308-1315. doi:10.1111/irv.12054 23176127 PMC4634266

[5] Bayham J, Fenichel EP. Impact of school closures for COVID-19 on the US health-care workforce and net mortality: a modelling study. Lancet Public Health. 2020;5(5):e271-e278. doi:10.1016/S2468-2667(20)30082-7 32251626 PMC7270508

[6] Cauchemez S, Van Kerkhove MD, Archer BN, . School closures during the 2009 influenza pandemic: national and local experiences. BMC Infect Dis. 2014;14:207. doi:10.1186/1471-2334-14-207 24739814 PMC4021091

[7] Chin ET, Huynh BQ, Lo NC, Hastie T, Basu S. Projected geographic disparities in healthcare worker absenteeism from COVID-19 school closures and the economic feasibility of child care subsidies: a simulation study. BMC Med. 2020;18(1):218. doi:10.1186/s12916-020-01692-w 32664927 PMC7360472

[8] Hiraoka D, Tomoda A. Relationship between parenting stress and school closures due to the COVID-19 pandemic. Psychiatry Clin Neurosci. 2020;74(9):497-498. doi:10.1111/pcn.13088 32779846 PMC7323183

[9] Kavanagh AM, Mason KE, Bentley RJ, . Leave entitlements, time off work and the household financial impacts of quarantine compliance during an H1N1 outbreak. BMC Infect Dis. 2012;12:311. doi:10.1186/1471-2334-12-311 23164090 PMC3533824

[10] Kim SJ, Lee S, Han H, Jung J, Yang SJ, Shin Y. Parental mental health and children’s behaviors and media usage during COVID-19-related school closures. J Korean Med Sci. 2021;36(25):e184. doi:10.3346/jkms.2021.36.e184 34184439 PMC8239422

[11] Kishida K, Tsuda M, Waite P, Creswell C, Ishikawa SI. Relationships between local school closures due to the COVID-19 and mental health problems of children, adolescents, and parents in Japan. Psychiatry Res. 2021;306:114276. doi:10.1016/j.psychres.2021.114276 34798486 PMC8585496

[12] Collins C, Landivar LC, Ruppanner L, Scarborough WJ. COVID-19 and the gender gap in work hours. Gend Work Organ. 2020. doi:10.1111/gwao.12506 32837019 PMC7361447

[13] Adams-Prassl A, Boneva T, Golin M, Rauh C. Inequality in the impact of the coronavirus shock: evidence from real time surveys. J Pub Econ. 2020;189:104245. doi:10.1016/j.jpubeco.2020.104245

[14] Kearny A, Hamel LMB. Mental health impact of the COVID-19 pandemic: an update. Accessed November 7, 2022. https://www.kff.org/coronavirus-covid-19/poll-finding/mental-health-impact-of-the-covid-19-pandemic/

[15] Njoku A, Ahmed Y, Bolaji B. Police brutality against Blacks in the United States and ensuing protests: implications for social distancing and Black health during COVID-19. J Hum Behav Soc Environ. 2021;31(1-4):262-270. doi:10.1080/10911359.2020.1822251

[16] Miu AS, Moore JR. Behind the masks: experiences of mental health practitioners of color during the COVID-19 pandemic. Acad Psychiatry. 2021;45(5):539-544. doi:10.1007/s40596-021-01427-w 33660237 PMC7929548

[17] Lopez PJ, Neely AH. Fundamentally uncaring: the differential multi-scalar impacts of COVID-19 in the U.S. Soc Sci Med. 2021;272:113707. doi:10.1016/j.socscimed.2021.113707 33517126 PMC8724555

[18] Shim RS, Starks SM. COVID-19, structural racism, and mental health inequities: policy implications for an emerging syndemic. Psychiatr Serv. 2021;72(10):1193-1198. doi:10.1176/appi.ps.202000725 33622042

[19] Dongarwar D, Ajewole VB, Oduguwa E, . Role of social determinants of health in widening maternal and child health disparities in the era of COVID-19 pandemic. Int J MCH AIDS. 2020;9(3):316-319. doi:10.21106/ijma.398 32765962 PMC7397329

[20] Fitzpatrick KM, Drawve G, Harris C. Facing new fears during the COVID-19 pandemic: the state of America’s mental health. J Anxiety Disord. 2020;75:102291. doi:10.1016/j.janxdis.2020.102291 32827869 PMC7425672

[21] Tai DBG, Shah A, Doubeni CA, Sia IG, Wieland ML. The disproportionate impact of COVID-19 on racial and ethnic minorities in the United States. Clin Infect Dis. 2021;72(4):703-706. doi:10.1093/cid/ciaa815 32562416 PMC7337626

[22] Mercado M, Wachter K, Schuster RC, . A cross-sectional analysis of factors associated with stress, burnout and turnover intention among healthcare workers during the COVID-19 pandemic in the United States. Health Soc Care Community.2022;30(5):e2690-e2701. doi:10.1111/hsc.13712 35037346

[23] McKnight-Eily LR, Okoro CA, Strine TW, . Racial and ethnic disparities in the prevalence of stress and worry, mental health conditions, and increased substance use among adults during the COVID-19 pandemic—United States, April and May 2020. MMWR Morb Mortal Wkly Rep. 2021;70(5):162-166. doi:10.15585/mmwr.mm7005a3 33539336 PMC7861483

[24] Hall LR, Sanchez K, da Graca B, Bennett MM, Powers M, Warren AM. Income differences and COVID-19: impact on daily life and mental health. Popul Health Manag. 2021;25(3):384-391. doi:10.1089/pop.2021.0214 34652228

[25] Gillman MW, Blaisdell CJ. Environmental influences on child health outcomes, a research program of the National Institutes of Health. Curr Opin Pediatr. 2018;30(2):260-262. doi:10.1097/MOP.0000000000000600 29356702 PMC6020137

[26] Romano ME, Buckley JP, Elliott AJ, Johnson CC, Paneth N, Program Collaborators for Environmental Influences on Child Health Outcomes. SPR perspectives: scientific opportunities in the environmental influences on Child Health Outcomes Program. Pediatr Res. 2021. doi:10.1038/s41390-021-01577-5 PMC8145190 34035428

[27] LeWinn KZ, Caretta E, Davis A, Anderson AL, Oken E, Program collaborators for Environmental influences on Child Health Outcomes. SPR perspectives: environmental influences on Child Health Outcomes (ECHO) Program: overcoming challenges to generate engaged, multidisciplinary science. Pediatr Res. 2021. doi:10.1038/s41390-021-01598-0 PMC8204620 34131290

[28] Environmental Influences on Child Health Outcomes (ECHO) Program. PhenX Toolkit ECHO COVID-19 Questionnaires. 2020. Accessed February 26, 2022. https://www.phenxtoolkit.org/covid19/source

[29] Margolis A, Algermissen M, Herbstman J, . Acute stress questions for ECHO COVID-19 survey. PsychArchives. 2021. Accessed November 8, 2022. https://www.psycharchives.org/en/item/d373a892-32e8-49f3-8c2b-9038f36c3235

[30] American Psychiatric Association. Diagnostic and statistical manual of mental disorders (5th ed.). 2013. American Psychological Association.

[31] Forgy EW. Cluster analysis of multivariate data—efficiency vs interpretability of classifications. Biometrics. 1965;21(3):768.

[32] Maechler M, Rousseeuw P, Struyf A, Hubert M. K H. cluster: cluster analysis basics and extensions. R package version 2.1.2. https://CRAN.R-project.org/package=cluster. 2021.

[33] de Amorim RC, Hennig C. Recovering the number of clusters in data sets with noise features using feature rescaling factors. Inf Sci. 2015;324:126-145. doi:10.1016/j.ins.2015.06.039

[34] Rousseeuw PJ. Silhouettes: a graphical aid to the interpretation and validation of cluster analysis. J Comput Appl Math. 1987;20:53-65. doi:10.1016/0377-0427(87)90125-7

[35] Chun H, Keleş S. Sparse partial least squares regression for simultaneous dimension reduction and variable selection. J R Stat Soc Series B Stat Methodol. 2010;72(1):3-25. doi:10.1111/j.1467-9868.2009.00723.x 20107611 PMC2810828

[36] Altman DG, Bland JM. Interaction revisited: the difference between two estimates. BMJ. 2003;326(7382):219. doi:10.1136/bmj.326.7382.219 12543843 PMC1125071

[37] Werchan DM, Hendrix CL, Ablow JC, . Behavioral coping phenotypes and associated psychosocial outcomes of pregnant and postpartum women during the COVID-19 pandemic. Sci Rep. 2022;12(1):1209. doi:10.1038/s41598-022-05299-4 35075202 PMC8786860

[38] Kinser PA, Jallo N, Amstadter AB, . Depression, anxiety, resilience, and coping: the experience of pregnant and new mothers during the first few months of the COVID-19 pandemic. J Womens Health (Larchmt). 2021;30(5):654-664. doi:10.1089/jwh.2020.8866 33844945 PMC8182651

