# Supplementary material for: COVID-19 Pandemic Experiences and Symptoms of Pandemic-Associated Traumatic Stress Among Mothers in the US
Source: JAMA Netw Open. 2022 Dec 16;5(12):e2247330. doi: 10.1001/jamanetworkopen.2022.47330 (PMC9856510; doi:10.1001/jamanetworkopen.2022.47330)
Supplement: Supplement 1. — eTable 1. Description of ECHO Cohorts Included in Analysis eFigure 1. Flowchart of Study Participants and Survey Selection eAppendix. Survey eTable 2. Secondary Pandemic-Associated Traumatic Stress Outcomes in Mothers of ECHO Children by Cluster and Overall eTable 3. Association Between Pandemic-Associated Hardships/Daily Life Changes, Coping Strategies, and Behavior Changes and Pandemic-Associated Traumatic Stress Total Sum Score Among Mothers of ECHO Children eTable 4. Association Between Pandemic-Associated Stressors, Coping Strategies, and Behavior Changes and Pandemic-Associated Traumatic Stress Symptoms Categories eMethods eFigure 2. Optimal Number of Clusters by Total Within Sum of Squares (Panel A) and Silhouette Width (Panel B) eTable 5. Pandemic-Associated Hardships, Coping Strategies and Behavior Changes By Cluster and Overall Reported by ECHO Mothers eFigure 3. Number of Total Significant Symptoms of Pandemic-Associated Traumatic Stress by Cluster in Mothers of ECHO Children eReferences [file jamanetwopen-e2247330-s001.pdf]

Supplemental Online Content

Bastain TM, Knapp EA, Law A, Environmental Influences on Child Health Outcomes Program Collaborators, et al. COVID-19 pandemic experiences and symptoms of pandemic-associated traumatic stress among mothers in the US. *JAMA Netw Open*. 2022;5(12):e2247330. doi:10.1001/jamanetworkopen.2022.47330

eTable 1. Description of ECHO Cohorts Included in Analysis

eFigure 1. Flowchart of Study Participants and Survey Selection

eAppendix. Survey

eTable 2. Secondary Pandemic-Associated Traumatic Stress Outcomes in Mothers of ECHO Children by Cluster and Overall

eTable 3. Association Between Pandemic-Associated Hardships/Daily Life Changes, Coping Strategies, and Behavior Changes and Pandemic-Associated Traumatic Stress Total Sum Score Among Mothers of ECHO Children

eTable 4. Association Between Pandemic-Associated Stressors, Coping Strategies, and Behavior Changes and Pandemic-Associated Traumatic Stress Symptoms Categories

eMethods

eFigure 2. Optimal Number of Clusters by Total Within Sum of Squares (Panel A) and Silhouette Width (Panel B)

eTable 5. Pandemic-Associated Hardships, Coping Strategies and Behavior Changes By Cluster and Overall Reported by ECHO Mothers

eFigure 3. Number of Total Significant Symptoms of Pandemic-Associated Traumatic Stress by Cluster in Mothers of ECHO Children

eReferences

This supplemental material has been provided by the authors to give readers additional information about their work.

eTable 1. Description of ECHO Cohorts Included in Analysis

Names	N	Description
ECHO in Puerto Rico (PROTECT)	16	Cohort consists of pregnant women recruited from 5 OB/GYN clinics in Puerto Rico and their offspring. Aim of study is To understand environmental exposures and their effects on birth outcomes and children's neurodevelopmental health in Puerto Rico.
Prematurity and Respiratory Outcomes Program (DINE-PROP)	75	Cohort consists of preterm infants ≤7 days old and gestational age (GA) between 23-<29 weeks. Aim of study is to identify clinical, physiologic, and biochemical markers during NICU hospitalization that can predict respiratory morbidity through 1 year of age.
Trial of Late Surfactant (DINE-TOLSURF)	37	Cohort consists of preterm infants ≤ 28 weeks GA intubated and mechanically ventilated at 7-14 days with plan to treat with inhaled nitric oxide. Aim of the study is to examine the effect of late doses of surfactant on bronchopulmonary dysplasia (BPD) at 36 weeks postmenstrual age (PMA) in preterm infants receiving inhaled nitric oxide.
NICU Hospital Exposures and Long-Term Health (DINE-NICUHEALTH)	48	Cohort consists of very low birth weight infants recruited upon admission to the NICU at a New York City hospital and their caregivers. Aim of study is to identify and assess the impact of NICU-based chemical and non-chemical environmental exposures on neurobehavioral outcomes through early childhood
Preterm Erythropoietin Neuroprotection Trial (DINE-PENUT)	27	Cohort population consists of infants 24 hours of age or less and between 24-<28 weeks GA admitted to the NICU. Aim of this study is to test the efficacy of erythropoietin (Epo) for the neuroprotection of extremely premature infants and assess whether early high dose Epo improves survival without neurodevelopmental impairment.
Family Life Project (FLP)	473	Cohort consists of population-based primary caregiver-child dyads (and secondary caregiver when available), with over-sampling for poverty and African American participants, in predominantly low-income, non-urban counties recruited at birth from 1 of 7 hospitals in Pennsylvania or North Carolina. Aim of this study is to investigate associations between psychosocial early life stress and neurodevelopment, including executive function, emotion regulation, language development, school achievement, and attention deficit/hyperactivity disorder (ADHD).
43rd Multicenter Airway Research Collaboration (MARC-43)	82	Serves as a cohort of healthy "controls" for the MARC-35 cohort, an ongoing, 17-center cohort comprised of 1016 infants initially hospitalized for bronchiolitis, of whom 921 are in long-term follow-up. Comprised of two groups: a) 120 healthy infants who were enrolled between November 2013 through May 2014 from a primary care group practice at Massachusetts General Hospital (MGH); and b) an additional 600 healthy infants from four U.S. sites that participated in MARC-35. Primary outcomes are recurrent wheezing by age 3 years, physician-diagnosis of asthma by age 5 years, and asthma diagnosis and phenotypes at 6 years by exam.

Healthy Start	184	Cohort consists of Colorado community sample of mother-child dyads recruited in infancy from obstetrics clinics at a university hospital and by word of mouth, as well as medical university employees. Aim is to understand the contribution of metabolic and behavioral factors during pregnancy to the development of childhood obesity, insulin resistance, and inflammatory markers.
BAMBAM	103	Cohort consists of Children recruited between 3 months to 12 years of age from the general population recruited in pediatrician offices and via online webpages, radio advertisements, and flyers in Providence, RI. Aims are to examine typical brain development, including how brain growth is altered by specific pre- and post-natal environmental or genetic factors; how patterns of brain growth are associated with, and predictive of, emerging cognitive and behavioral abilities; and how these brain-behavior relationships are influenced by modifiable factors experienced throughout childhood
MINNIE	87	Cohort consists of Children recruited between 3 months to 12 years of age from the general population recruited in pediatrician offices and via online webpages, radio advertisements, and flyers in Providence, RI. Aims are to examine typical brain development, including how brain growth is altered by specific pre- and post-natal environmental or genetic factors; how patterns of brain growth are associated with, and predictive of, emerging cognitive and behavioral abilities; and how these brain-behavior relationships are influenced by modifiable factors experienced throughout childhood
Boricua Youth Study (BYS)	97	Cohort consists of Latinx youth identifying as being of Puerto Rican background recruited in New York, NY and Puerto Rico during childhood and their caregivers. Aim of study is to examine key factors that influence intergenerational socioeconomic disadvantage and the impact of adversity on child neurodevelopmental health and functioning
Atlanta ECHO Cohort of Emory University	116	Cohort consists of African American women recruited from two prenatal clinics in Atlanta, GA and their offspring. Aim is to identify intrauterine and early childhood environmental exposures and risk pathways contributing to children's neurodevelopmental deficits and obesity.
Safe Passage Study (PASS)	1038	Cohort is made up of White and American Indian women recruited in pregnancy from participating obstetrics and gynecology clinics and other entities (e.g., the Supplemental Nutrition Program for Women, Infants, and Children) in South Dakota. Aim is to investigate the relationship between prenatal alcohol exposure, stillbirth, and sudden infant death syndrome.

© 2022 Bastain TM et al. *JAMA Network Open*

Pregnancy Environment and Lifestyle Study (PETALS)	283	Cohort consists of women identified as pregnant via hospital records from 4 California hospitals recruited before 10 weeks gestation via telephone and their offspring. Aim is to examine whether BPA levels are associated with gestational diabetes and growth development.
Kaiser Permanente Research Bank (KPRB)	173	Cohort consists of women identified as pregnant via hospital records from 4 California hospitals recruited before 10 weeks gestation via telephone and their offspring. Aim is to examine whether BPA levels are associated with gestational diabetes and growth development.
Microbes, Allergy, Asthma & Pets Study (MAAP)	19	Cohort consists of pregnant women recruited from 2 hospital systems in Michigan and their offspring, half of whom lived in households with dogs and half of whom live in pet-free households. Aim is to investigate child immune development and intestinal microbiota for children growing up with dogs compared to those in pet-free households.
Wisconsin Infant Cohort Study (WISC)	60	A longitudinal birth cohort started in 2013 in rural Wisconsin comprised of Healthy neonates 34 weeks' gestation and parents/guardians. There are two study arms: farm exposed versus non-farm exposed. The primary outcome is to determine respiratory virus illness burden during the first two years of life between the two study arms. Secondary outcomes include defining differences in immune maturation, environmental microbial exposures, and expression of allergic diseases.
Urban Environment and Childhood Asthma (URECA)	122	Cohort consists of children whose biological mothers were recruited in pregnancy from 4 hospitals in St. Louis, Boston, Baltimore, and New York; who have a parental history of asthma, allergic rhinitis, or eczema; and who reside in census tracts with at least 20% of residents with income below the poverty level. Aim is to examine the impact of living in inner-city environments on the development of asthma and identifying more effective treatments for high-asthma risk group with a lens towards future prevention effort
Infant Susceptibility to Pulmonary Infections and Asthma Following RSV Exposure (INSPIRE)	81	Cohort consists of children born between June 1 – December 31 of the recruitment years to ensure they were on average 6 months of age or less during their first respiratory syncytial virus (RSV) season recruited from 11 pediatric clinics in central Tennessee. Aim is to understand how respiratory infections during the first year of life impact health later in childhood.
Wayne County Health Environment Allergy and Asthma (WHEALS)	111	Population-based birth cohort study being conducted in Detroit, MI and Western Wayne County MI suburbs. Aims is to examine the relationships between early life exposures such as pets, infections, and the environmental and infant gut microbiome in relationship to the development of allergic diseases.

© 2022 Bastain TM et al. *JAMA Network Open*

Childhood Allergy and the Neonatal Environment (CANOE)	7	A diverse multicenter birth cohort of families recruited from Detroit, MI, Madison, WI, Nashville, TN, and St. Louis, MO where all families have at least one parent with allergic disease or asthma. Aims is to identify prenatal and early life exposures that are related to the development of allergies and wheezing illnesses in the first two years of life
Maternal and Developmental Risks from Environmental and Social Stressors (MADRES)	270	The MADRES pregnancy cohort is comprised of predominantly low-income Hispanic/Latina women and children in urban Los Angeles. The cohort was established to investigate the cumulative effects of prenatal and early life environmental exposures in combination with the effects of social stressors to affect the risk of early childhood and maternal health outcomes among environmentally- and socioeconomically-burdened communities.
Revisiting CHildhood Autism Risk from Genes and the Environment Study (ReCHARGE)	129	Cohort is comprised of children ages 2-5 years with a diagnosis of autism spectrum disorder (ASD) or another developmental disorder identified from the California Department of Developmental Services list of individuals receiving services for ASD or other neurobehavioral delays, from regional service coordination centers, or referrals from clinics or self-referral; Control children were sampled from birth files matched on demographics to the ASD sample. Aim is to identify environmental chemical and non-chemical stressors and resiliency factors associated with neurodevelopmental outcomes in childhood and adolescence.
Pittsburgh Girls Study (PGS)	52	A longitudinal study, sampled from the population of the City of Pittsburgh (disadvantaged neighborhoods were over-sampled) to examine the development of behavioral and emotional problems in a population-based sample of Black and White girls living in an urban environment.
New Hampshire Birth Cohort Study (NHBCS)	1051	Cohort consists of pregnant women recruited from 5 prenatal clinics in the rural New Hampshire and their offspring. Aim is to investigate the impact of in-utero and early life environmental contaminants (e.g., arsenic) on maternal prenatal health, birth outcomes, growth, and neurodevelopment.
Conditions Affecting Neurocognitive Development and Learning in Early Childhood (CANDLE)	328	Cohorts consist of a majority African American sample of mother-child dyads recruited in pregnancy in Memphis, TN from safety net obstetrical clinics and local OB/GYN partners, television and radio advertisements, and directed mailings. Aims is to understand early-life predictors of child socioemotional and neurocognitive development.

© 2022 Bastain TM et al. *JAMA Network Open*

The Global Alliance to Prevent Prematurity and Stillbirth (GAPPS)	26	Cohort consists of pregnant women or women who delivered within 7 days on enrollment from three hospitals in Washington state with racial/ethnic and socioeconomically diverse populations, high pre-term birth rates, and geographic and environmental differences. Aim is to examine how chemical and non-chemical exposures during pregnancy relate to placental gene expression and child neurodevelopmental health and functioning.
Neonatal Neurobehavior and Outcomes in Very Preterm Infant (ECHO-NOVI)	217	Cohort consists of Infants both <30 weeks gestational age in 8 NICUs across the US (Michigan, Hawaii, North Carolina, California, and Rhode Island) and their caregivers. Aim is to determine which infants born <30 weeks gestational age are at greatest risk for impaired development based on the NICU Network Neurobehavioral Scale (NNS) and medical risk score and the role of the postnatal environment in moderating risks on early childhood developmental outcomes.
Early Growth and Development Study (EGDS-CI)	169	Cohort consists of biological parents, adoptive parents, and their children recruited at the time of birth through adoption agencies in Oregon, Washington, Texas, Maryland, Pennsylvania, Virginia, New Jersey, California, Utah, Minnesota, Illinois, and Florida. The primary study aims are to examine how family, peer and contextual processes affect children's adjustment, and to examine their interplay (mediation, moderation) with genetic influences.
Early Growth and Development Study (EGDS-CII) 2	90	Cohort consists of biological parents, adoptive parents, and their children recruited at the time of birth through adoption agencies in Oregon, Washington, Texas, Maryland, Pennsylvania, Virginia, New Jersey, California, Utah, Minnesota, Illinois, and Florida. The primary study aims are to examine how family, peer and contextual processes affect children's adjustment, and to examine their interplay (mediation, moderation) with genetic influences.
Early Parenting of Children (EPoCh)	33	Sample consists of 200 sibling pairs, recruited from one site in Oregon, in which one sibling was reared from birth with an adoptive family, and the other sibling was reared from birth by the biological mother of the sibling pair. Utilizes a naturalistic human cross-fostering design to advance the understanding of childhood pathways to risk for drug use by identifying nuances in the rearing environment that are associated with child risk behaviors and competencies.
Navajo Birth Cohort Study (NBCS) / Understanding Risk Gradients from Environment on Native American Child Health	160	Sample is comprised of Native American mothers who have lived on Navajo Nation or within adjacent community for at least 5 years and their children, recruited from sites in New Mexico and Arizona. Aims to evaluate the influence of prenatal and postnatal exposure to metals on birth, neurodevelopmental, immunological, and obesity outcomes.

© 2022 Bastain TM et al. *JAMA Network Open*

Trajectories (URGENT)		
Vitamin D Antenatal Asthma Reduction Trial (VDAART)	327	Cohort consists of children of women recruited prenatally from 3 OB/GYN sites in Boston, MA, St. Louis, MO, and San Diego, CA whose biological mother or father had a history of asthma, eczema, and/or allergic rhinitis. Aim is to examine the role of allergy, the environment, and genetics in developing asthma as well as investigating whether vitamin D levels in pregnancy prevent or reduce asthma, wheezing, or other allergic illnesses in childhood.
Vitamin C to Decrease Effects of Smoking in Pregnancy on Infant Lung Function (VCSIP)	185	Cohort consists of pregnant women who smoke receiving prenatal care at 3 clinics across the US (Indiana, Washington, Oregon) and their offspring. Aims is to characterize the epigenetic mechanisms underlying the effects of prenatal smoking on child lung health and to determine if the protective role of vitamin C supplementation in pregnancy on child pulmonary health extends to early adolescence.
In-Utero Smoke, Vitamin C, and Newborn Lung Function	94	Cohort is comprised of pregnant women who smoke receiving prenatal care at 3 clinics in Washington and Oregon and their offspring. Aim is to characterize the epigenetic mechanisms underlying the effects of prenatal smoking on child lung health and to determine if the protective role of vitamin C supplementation in pregnancy on child pulmonary health extends to early adolescence.
Kennedy Krieger – Baby Siblings Research Consortium (Kennedy Krieger-BSRC)	<5	Cohort consists of children whose older sibling has autism spectrum disorder (ASD) recruited from the general population primarily in Maryland from doctors, hospitals, universities, public libraries, faith-based sites, and parent support groups. Aim is to examine the role of genetic and environmental factors and their interactions on ASD-related neurodevelopmental outcomes.
University of California Davis – Baby Siblings Research Consortium (UCDavis)	20	Cohort is comprised of infants with older siblings with confirmed autism spectrum disorder (ASD) diagnosis and infants with at least one older sibling with no evidence of ASD or other neurodevelopmental disorder, recruited from a large database of children with neurodevelopmental disorders and typical development as well as local preschools, clinics, and other agencies in the Sacramento, CA area serving young children with ASD. Aim is to examine the role of genetic and environmental factors and their interactions on ASD-related neurodevelopmental outcomes

© 2022 Bastain TM et al. *JAMA Network Open*

Infant Brain Imaging Study (IBIS)	25	Cohort consists of infants with an older biological sibling with autism spectrum disorder (ASD) or control infants with at least one older, typically-developing sibling recruited from AutismSpeaks email blasts to North Carolina, Washington, and Pennsylvania as well as local autism events, pediatricians, and OB/GYNs. Aim is to examine developmental paths of infants at high risk for ASD, identify early developmental markers of individual ASD outcomes, and identify the risk and protective factors that account for individual differences.
Early Autism Risk Longitudinal Investigation (EARLI)	42	Cohort consists of pregnant women with a biological child with autism spectrum disorder (ASD) and the offspring of the current pregnancy, recruited from local community events, autism conferences, direct mail via autism service providers, and online and social media. Aim is to understand pre-, peri-, and postnatal ASD risk factors and risk biomarkers.
Rochester	82	Cohort consists of women recruited during the first trimester of pregnancy from clinics in Rochester, NY serving high psychosocial risk populations, oversampled for psychosocial stress. The aim is to examine if and how prenatal exposures “program” adaptive biological responses in the fetus and child and carry-forward effects on neurodevelopmental health and obesity, with a focus on inflammation as a biological mechanistic pathway.
Magee	155	Cohort consists of women recruited during the first trimester of pregnancy from clinics in Pittsburgh, PA serving high psychosocial risk populations, oversampled for childhood trauma, and their offspring. Aim is to examine if and how prenatal exposures “program” adaptive biological responses in the fetus and child and carry-forward effects on neurodevelopmental health and obesity, with a focus on inflammation as a biological mechanistic pathway.
Project Viva	483	Cohort is comprised of pregnant women receiving prenatal care at one of 8 urban and suburban clinics in and around Boston, MA and their offspring. Aim is to examine the effects of modifiable exposures (e.g., diet, behaviors, environment) during pregnancy and after birth to improve maternal and child health.
Extremely Low Gestational Age Newborn (ELGAN)	276	Cohort is made up of preterm infants (<28 weeks gestation) recruited from 1 of 15 US hospitals in North Carolina, Michigan, Illinois, Connecticut, and Massachusetts. Aim is to investigate neurodevelopmental outcomes (e.g., cerebral palsy, cognitive impairment, autism spectrum disorder, anxiety, depression, brain MRI abnormalities) in an extremely preterm population
Archive for Research on Child Health (ARCH)	421	Cohort consists of Pregnant women recruited during their first prenatal visit at 1 of 3 prenatal clinics in Lansing, MI. Aim is to examine pre- and perinatal environmental contaminants, nutritional factors, and inflammation in mother-infant dyads in the context of their social and psychological environment on child neurodevelopmental and obesity outcomes.

© 2022 Bastain TM et al. *JAMA Network Open*

Michigan Archive for Research in Child Health (MARCH)	378	Statewide probability sample of pregnant women in 20 prenatal care settings in Michigan to examine pre- and perinatal environmental contaminants, nutritional factors, and inflammation in mother-infant dyads in the context of their social and psychological environment on child neurodevelopmental and obesity outcomes
CCCEH: Sibling/Hermanos Cohort	45	A cohort of Dominican or African American low-income mothers and their offspring recruited at OB/GYN clinics in New York, NY. to investigate the associations between prenatal exposure to environmental toxic pollutants and adverse childhood outcomes, including reduced IQ, ADHD, and obesity.
CCCEH: Fair Start	161	A cohort of Dominican or African American low-income mothers and their offspring recruited at OB/GYN clinics in New York, NY. to investigate the associations between prenatal exposure to environmental toxic pollutants and adverse childhood outcomes, including reduced IQ, ADHD, and obesity.
Illinois Kids Development Study (IKIDS)	79	Cohort of pregnant women recruited from 2 OB/GYN clinics in southern Illinois and their offspring to examine the unique and combined impact of prenatal exposure to endocrine disrupting chemicals and maternal stress on child neurodevelopmental health and functioning.
Chemicals in our Bodies (CIOB)	216	Cohort consists of pregnant women recruited from 2 hospitals in San Francisco, CA and their offspring and aims to examine the unique and combined impact of prenatal exposure to endocrine disrupting chemicals and maternal stress on child neurodevelopmental health and functioning.
National Children's Study – IVS Salt Lake County (NCS-IVS-SLC) + Associate	64	A population-based sample of pregnant women, or women planning pregnancy, enrolled from Salt Lake County, Utah that examines the environmental influences on children's health and development. The "environment" was defined broadly to include physical environment (neighborhoods and communities, housing radiation); biological (infectious agents, glucose metabolism, diet) and chemical factors (pesticides, air pollution, pharmaceuticals); geography; psychosocial environment (social networks, socioeconomic status, education, behavioral, family, and cultural influences); and genetics (gene-environment and gene-gene interactions).

© 2022 Bastain TM et al. *JAMA Network Open*

National Children's Study – Alternate Recruitment Strategy Cache County (NCS-ARS-Cache) + Associate	122	A population-based sample of pregnant women, or women planning pregnancy, enrolled from Cache County, Utah that examines the environmental influences on children's health and development. The "environment" was defined broadly to include physical environment (neighborhoods and communities, housing radiation); biological (infectious agents, glucose metabolism, diet) and chemical factors (pesticides, air pollution, pharmaceuticals); geography; psychosocial environment (social networks, socioeconomic status, education, behavioral, family, and cultural influences); and genetics (gene-environment and gene-gene interactions).
Home Observation of Periconceptional Exposures – Utah (HOPE-Utah) + Associate	49	Prospective, preconception cohort of heterosexual couples, in Salt Lake City Utah, trying to conceive within three months of study enrollment to evaluate the association between prospectively measured male and female environmental exposures (BPA and disinfection-by-products) and semen quality and time to pregnancy.
Nulliparous Pregnancy Outcomes Study: Monitoring Mothers-to-be (NuMoM2b-Utah) + Associate	97	A prospective cohort study of nulliparous understudied at-risk women in Salt Lake City, Utah to evaluate the underlying, interrelated mechanisms of several common adverse pregnancy outcomes, which can be unpredictable in women who have little or no pregnancy history, to help guide treatment.
Time to Pregnancy – Utah (TTP-Utah) + Associate	36	Women with no prior pregnancy planning to conceive in Salt Lake City, Utah to assess the impact of the Creighton Model Fertility Care System (CrMS) on time to pregnancy (TTP), via per-cycle pregnancy rates (fecundability)
Baby Affect & Behavior Study (BABY-Utah)	30	Cohort of pregnant women in Salt Lake City, Utah to examine physiological and epigenetic mechanisms by which maternal prenatal emotion dysregulation could affect newborn neurobehavioral outcomes.
The NYU Children's Health and Environment Study (NYU CHES)	878	A longitudinal study of pregnant women recruited from prenatal clinics at NYU Langone Medical Center study sites that collects, processes, and stores maternal and child biological samples and data to help facilitate research on fetal growth and development
Asthma Coalition on Community, Environment & Social Stress (ACCESS)	<5	Cohort of pregnant women receiving prenatal care at 1 of 6 sites (hospital, clinics affiliated with Women, Infants, and Children [WIC] programs, community health centers) in and around Boston, MA chosen for their heterogeneity in sociodemographic and racial/ethnic characteristics, and their offspring that aims to examine how prenatal and childhood chemical and non-chemical pro-oxidant environmental factors impact child neurodevelopment, stress regulation, and respiratory health

© 2022 Bastain TM et al. *JAMA Network Open*

PRogramming of Intergenerational Stress Mechanisms (PRISM)	395	Cohort of pregnant women recruited from prenatal clinics at hospitals and community health centers in Boston, MA and New York, NY, and their offspring to examine how prenatal and childhood chemical and non-chemical pro-oxidant environmental factors impact child neurodevelopment, stress regulation, and respiratory health.
First 1000 Days Study/GMU ECHO Cohort	600	This study, initially nested within a healthcare system in Northern Virginia, is now followed under George Mason University. The initial cohort was started in 2012 and launched as a longitudinal pregnancy/ birth cohort framework. This study accrued 5,000 children within a highly diverse (ethnically and socio-economically) community. Because ICLS is a genetics study, consent for whole-genome sequencing (WGS) was obtained and was done on approximately 1500 families.
The Infant Development and the Environment Study (TIDES)	395	Cohort consists of pregnant women recruited from prenatal clinics in Minnesota, New York, California, and Washington, and their offspring. Aims to examine in utero phthalate exposure in relation to sex-specific genital outcomes in infants and neurobehavior in children.
Columbia Center for Children's Environmental Health (CCCEH) / Mothers and Newborns (M and N)	14	Cohort comprised of Dominican or African American low-income mothers and their offspring recruited at OB/GYN clinics in New York, New York. Aims to investigate the associations between prenatal exposure to environmental toxic pollutants and adverse childhood outcomes, including reduced IQ, ADHD, and obesity.
Columbia Center for Children's Environmental Health	35	Dominican or African American low-income mothers and their offspring recruited at OB/GYN clinics in New York, New York.

© 2022 Bastain TM et al. *JAMA Network Open*

eFigure 1. Flowchart of Study Participants and Survey Selection

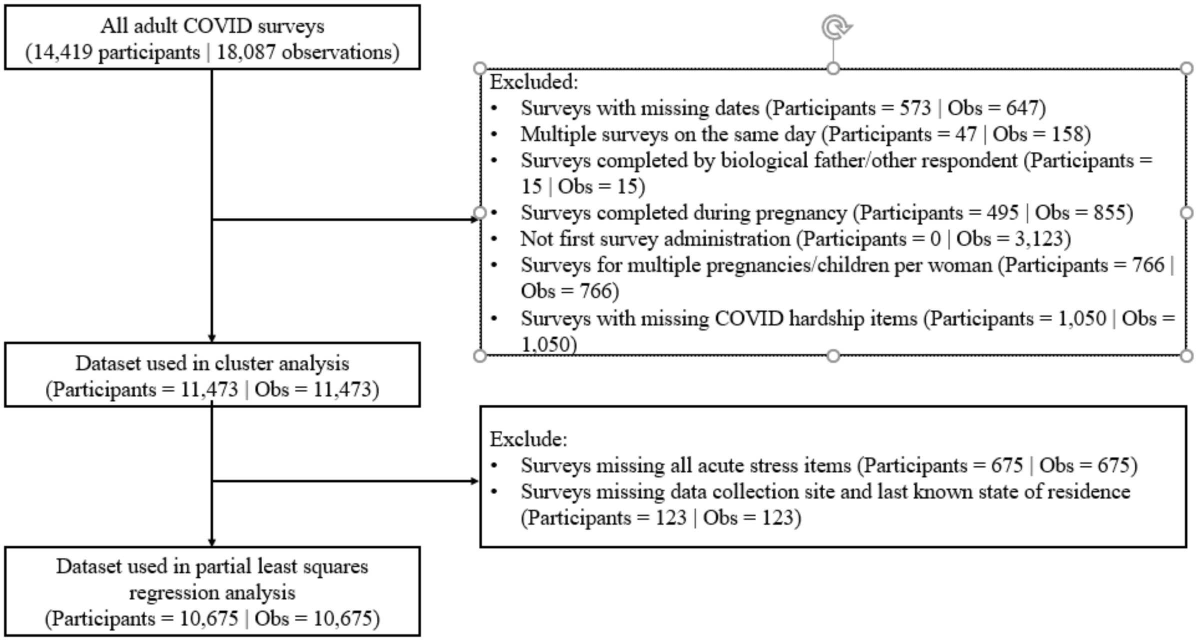

© 2022 Bastain TM et al. *JAMA Network Open*

eAppendix. Survey

ECHO Environmental influences on Child Health Outcomes A program supported by the NIH	**COVID-19 Questionnaire 2 – Adult Primary Version**<br>ECHO-wide Cohort Version 01.30 / August 6, 2020				**Form C19-2-aPV**<br>Page 1 of 9
**COHORT ID**	**SITE ID**	**PARTICIPANT ID**	**PIN**	**COHORT VISIT ID**	**FORM COMPLETED**
__ __ __ __ __ __	__ __ __ __ __	____________	__ __ __	___________	__ __/__ __/__ __ __ __<br>mm dd yyyy

**ECHO LIFE STAGE**		**RESPONDENT**	
☐01 Prenatal	☐02 Perinatal	☐01 Participant	☐02 Biological Mother
☐03 Infancy	☐04 Early Childhood	☐03 Biological Father	☐04 Other Respondent → Code: __ __
☐05 Middle Childhood	☐06 Adolescence		

STUDY STAFF INSTRUCTION: This form should be completed by the pregnant woman enrolled in an ECHO cohort during the prenatal life stage and by the primary caregiver of a child enrolled in an ECHO cohort during the infancy, early childhood, middle childhood, and adolescence life stages. In the prenatal life stage, the pregnant woman's ID should be used in the header for the participant ID. In all other life stages, the child's ID should be used in the header for the participant ID.

INSTRUCTIONS:

This form has 4 sections:

- *Section A: COVID-19 Infection*
- *Section B: Impacts of the COVID-19 Outbreak on You*
- *Section C: Impacts of the COVID-19 Outbreak on Pregnancy – Current*
- *Section D: Impacts of the COVID-19 Outbreak on Pregnancy – Recall*

Please complete Sections A and B. If you enrolled in ECHO during pregnancy and are currently pregnant, please also complete Section C. If you enrolled in ECHO during pregnancy and the pregnancy ended after February 28, 2020, please also complete Section D.

These questions are about your experience with COVID-19, or the coronavirus. For each question, do the best you can to remember the details requested.

© 2022 Bastain TM et al. *JAMA Network Open*

Section A. COVID-19 Infection

For the following questions, healthcare provider means a doctor, nurse practitioner, physician assistant or anyone you go to for medical care.

1. Has a healthcare provider ever told you that you have, or likely have, COVID-19 (Coronavirus)?

- ☐ 01 Yes
- ☐ 02 No

2. Which of the following symptoms have you had at any point in time since March 1, 2020? (***Mark all that apply***)

- ☐ 01 Fever or chills
- ☐ 02 Cough
- ☐ 03 Shortness of breath
- ☐ 04 Sore throat
- ☐ 05 Headache
- ☐ 06 Muscle or body aches
- ☐ 07 Runny nose
- ☐ 08 Fatigue or excessive sleepiness
- ☐ 09 Diarrhea, nausea, or vomiting
- ☐ 10 Loss of sense of smell or taste
- ☐ 11 Itchy/red eyes
- ☐ 12 None of the above ➔ ***skip to Section A, Question 3.***

2.a. Which of the following occurred as a result of your symptoms? (***Mark all that apply***)

- ☐ 01 I was kept overnight in a hospital because a healthcare provider thought I had COVID-19
- ☐ 02 I saw a healthcare provider in person, such as in a clinic, doctor's office, urgent care, or Emergency Room (ER)/Emergency Department (ED)
- ☐ 03 I spoke to a healthcare provider over the phone, by email, or online
- ☐ 04 I self-isolated or quarantined at home
- ☐ 05 None of the above

2.b. In the two weeks before you had symptoms, did you: (***Mark all that apply***)

- ☐ 01 Have contact with someone who tested positive for COVID-19
- ☐ 02 Have contact with someone who likely had COVID-19 (e.g., was not tested but had symptoms; was told by a healthcare provider that he/she likely had it)
- ☐ 03 Travel to a different state or country (please specify: __________________)
- ☐ 04 None of the above

© 2022 Bastain TM et al. *JAMA Network Open*

Section A. COVID-19 Infection (continued)

3. Have you had the nose swab test for the virus that causes COVID-19? (*Mark all that apply*)
   - ☐01 No, I never tried to get tested
   - ☐02 No, I tried to get tested but was not able to
   - ☐03 Yes, and I am waiting for the results
     - *If yes*➔ 3.a. When was the date of your most recent test? __ __ /__ __ __ __ (*mm* / *yyyy*)
   - ☐04 Yes, and the test showed that I do not have it ("**negative**" test)
     - *If yes* ➔ 3.b. When was the date of your most recent **negative** test? __ __ /__ __ __ __ (*mm* / *yyyy*)
   - ☐05 Yes, and the test showed that I do have it ("**positive**" test)
     - *If yes* ➔ 3.c. When was the date of your most recent **positive** test? __ __ /__ __ __ __ (*mm* / *yyyy*)

4. Have you had the mouth swab test for the virus that causes COVID-19? (*Mark all that apply*)
   - ☐01 No, I never tried to get tested
   - ☐02 No, I tried to get tested but was not able to
   - ☐03 Yes, and I am waiting for the results
     - *If yes* ➔ 4.a. When was the date of your most recent test? __ __ /__ __ __ __ (*mm* / *yyyy*)
   - ☐04 Yes, and the test showed that I do not have it ("**negative**" test)
     - *If yes* ➔ 4.b. When was the date of your most recent **negative** test? __ __ /__ __ __ __ (*mm* / *yyyy*)
   - ☐05 Yes, and the test showed that I do have it ("**positive**" test)
     - *If yes* ➔ 4.c. When was the date of your most recent **positive** test? __ __ /__ __ __ __ (*mm* / *yyyy*)

5. Have you had a blood test to see whether you already had the COVID-19 virus ("serology")? (*Mark all that apply*)
   - ☐01 No, I never tried to get tested
   - ☐02 No, I tried to get tested but was not able to
   - ☐03 Yes, and I am waiting for the results
     - *If yes* ➔ 5.a. When was the date of your most recent test? __ __ /__ __ __ __ (*mm* / *yyyy*)
   - ☐04 Yes, and the test showed that I did not have it ("**negative**" test)
     - *If yes* ➔ 5.b. When was the date of your most recent **negative** test? __ __ /__ __ __ __ (*mm* / *yyyy*)
   - ☐05 Yes, and the test showed that I did have it ("**positive**" test)
     - *If yes* ➔ 5.c. When was the date of your **positive** test? __ __ /__ __ __ __ (*mm* / *yyyy*)

6. Has anyone else living in your home had, or probably had, COVID-19?
   - ☐01 Yes
   - ☐02 No

© 2022 Bastain TM et al. *JAMA Network Open*

Section B. Impacts of the COVID-19 Outbreak on You

1. In what ways has the COVID-19 outbreak affected your overall healthcare? (***Mark all that apply***)

- ☐01 I did not go to healthcare appointments because I was concerned about entering my healthcare provider's office
- ☐02 My healthcare provider canceled appointments
- ☐03 My healthcare provider changed to phone or online visits
- ☐04 My healthcare provider told me to self-isolate or quarantine
- ☐05 None of these apply

2. Which of the following behaviors have you done less because of the COVID-19 outbreak? (***Mark all that apply***)

- ☐01 In-person contact with people inside the home (that is, you are quarantined separately from one or more family or household members)
- ☐02 In-person contact with family who live outside the home
- ☐03 In-person contact with friends
- ☐04 In-person contact with colleagues at work
- ☐05 In-person events in the community, including religious events
- ☐06 None of these apply

3. Which of the following behaviors have you changed because of the COVID-19 outbreak? (***Mark all that apply***)

- ☐01 Eat more home-cooked meals
- ☐02 Eat more takeout / delivered food
- ☐03 Get more physical exercise
- ☐04 Get less physical exercise
- ☐05 Spend more time outdoors in nature
- ☐06 Spend less time outdoors in nature
- ☐07 None of these apply

4. In what ways has the COVID-19 outbreak affected your work? (***Mark all that apply***)

- ☐01 I moved to working remotely or from home
- ☐02 I lost my job permanently
- ☐03 I lost my job temporarily, or was not told for how long
- ☐04 I got a new job
- ☐05 I reduced my work hours
- ☐06 I increased my work hours
- ☐07 My job put me at increased risk of getting COVID-19
- ☐08 I laid off employees
- ☐09 I did not have a paying job before the COVID-19 outbreak
- ☐10 None of these apply

© 2022 Bastain TM et al. *JAMA Network Open*

Section B. Impacts of the COVID-19 Outbreak on You (continued)

5. In what ways has the COVID-19 outbreak affected your spouse/partner's work? (*Mark all that apply*)
   - ☐00 Not applicable – I do not have a spouse/partner ➔ *If marked, skip to Section B, Question 6.*
   - ☐01 My spouse/partner moved to working remotely or from home
   - ☐02 My spouse/partner lost his/her job permanently
   - ☐03 My spouse/partner lost his/her job temporarily, or was not told for how long
   - ☐04 My spouse/partner got a new job
   - ☐05 My spouse/partner reduced his/her work hours
   - ☐06 My spouse/partner increased his/her work hours
   - ☐07 My spouse/partner's job put him/her at increased risk of getting COVID-19
   - ☐08 My spouse/partner laid off employees
   - ☐09 My spouse/partner did not have a paying job before the COVID-19 outbreak
   - ☐10 None of these apply

6. How has the COVID-19 outbreak affected your regular childcare? (*Mark all that apply*)
   - ☐01 I had difficulty arranging for childcare
   - ☐02 I had to pay more for childcare
   - ☐03 My spouse/partner or I had to change our work schedule to care for our children ourselves
   - ☐04 My regular childcare has not been affected by the COVID-19 outbreak
   - ☐05 I do not have a child in childcare.

7. What have been your greatest sources of stress from the COVID-19 outbreak? (*Mark all that apply*)
   - ☐01 Health concerns
   - ☐02 Financial concerns
   - ☐03 Impact on work
   - ☐04 Impact on your child
   - ☐05 Impact on your community
   - ☐06 Impact on family members
   - ☐07 Access to food
   - ☐08 Access to baby supplies (e.g., formula, diapers, wipes)
   - ☐09 Access to personal care products or household supplies
   - ☐10 Access to medical care, including mental health care
   - ☐11 Social distancing or being quarantined
   - ☐12 I am not stressed about the COVID-19 outbreak

© 2022 Bastain TM et al. *JAMA Network Open*

Section B. Impacts of the COVID-19 Outbreak on You (continued)

8. What have you done to cope with your stress related to the COVID-19 outbreak? (***Mark all that apply***)
   - ☐01 Meditation and/or mindfulness practices
   - ☐02 Talking with friends and family (e.g., by phone, text, or video)
   - ☐03 Engaging in more family activities (e.g., games, sports)
   - ☐04 Increased television watching or other "screen time" activities (e.g., video games, social media)
   - ☐05 Eating more often, including snacking
   - ☐06 Increasing time reading books, or doing activities like puzzles and crosswords
   - ☐07 Drinking alcohol
   - ☐08 Using tobacco (e.g., smoking, vaping)
   - ☐09 Using marijuana (e.g., vaping, smoking, eating) or cannabidiol (CBD)
   - ☐10 Talking to my healthcare providers more frequently, including mental healthcare provider (e.g., therapist, psychologist, counselor)
   - ☐11 Volunteer work
   - ☐12 I have not done any of these things to cope with the COVID-19 outbreak

9. Please indicate the extent to which you view the COVID-19 outbreak as having either a positive or negative impact on your life.
   - ☐01 Extremely negative
   - ☐02 Moderately negative
   - ☐03 Somewhat negative
   - ☐04 No impact
   - ☐05 Slightly positive
   - ☐06 Moderately positive
   - ☐07 Extremely positive

10. Since becoming aware of the COVID-19 outbreak, how often have you felt happy and satisfied with your life?
    - ☐01 Not at all
    - ☐02 Rarely
    - ☐03 Sometimes
    - ☐04 Often
    - ☐05 Very often

© 2022 Bastain TM et al. *JAMA Network Open*

Section B. Impacts of the COVID-19 Outbreak on You (continued)

For rows 11.a through 11.i below, please mark 'Not at all', 'Rarely', 'Sometimes', 'Often', or 'Very often' for how often you have had the experience since becoming aware of the COVID-19 outbreak.

**11.** Since becoming aware of the COVID-19 outbreak, how often have you …	Not at all	Rarely	Sometimes	Often	Very often
a. had difficulty sleeping	☐01	☐02	☐03	☐04	☐05
b. startled easily	☐01	☐02	☐03	☐04	☐05
c. had angry outbursts	☐01	☐02	☐03	☐04	☐05
d. felt a sense of time slowing down	☐01	☐02	☐03	☐04	☐05
e. felt in a daze	☐01	☐02	☐03	☐04	☐05
f. tried to avoid thoughts and feelings about COVID-19	☐01	☐02	☐03	☐04	☐05
g. tried to avoid reading or watching information about COVID-19	☐01	☐02	☐03	☐04	☐05
h. had distressing dreams about COVID-19	☐01	☐02	☐03	☐04	☐05
i. been distressed when I see something that reminds me of COVID-19	☐01	☐02	☐03	☐04	☐05

12. To route you through the remaining questions, please mark whether:

- ☐01 you enrolled in ECHO during pregnancy and are currently pregnant → ***If marked, skip to Section C.***
- ☐02 you enrolled in ECHO during pregnancy and the pregnancy ended after February 28, 2020 → ***If marked, skip to Section D.***
- ☐03 neither of the above → ***If marked, skip to END.***

© 2022 Bastain TM et al. *JAMA Network Open*

Section C. Impacts of the COVID-19 Outbreak on Pregnancy - Current

The following questions are about your current pregnancy.

1. Which of the following changes have you experienced as a result of the COVID-19 outbreak? (***Mark all that apply***)

- ☐ 01 I changed from planning a vaginal birth to a C-section
- ☐ 02 My planned C-section or labor induction was changed
- ☐ 03 I changed from planning a home birth to planning a hospital birth
- ☐ 04 I changed from planning a hospital birth to planning a home birth
- ☐ 05 My healthcare provider canceled some or all of my prenatal visits
- ☐ 06 I had more prenatal visits
- ☐ 07 My prenatal visits changed from in-person to phone or telemedicine/video
- ☐ 08 Nothing changed in my prenatal care or birth plan

2. In general, how distressed are you about **changes to your prenatal care** due to the COVID-19 outbreak?

- ☐ 01 Not at all
- ☐ 02 Mildly
- ☐ 03 Moderately
- ☐ 04 Extremely

3. How has the support you receive from your **prenatal care provider(s)** changed due to the COVID-19 outbreak?

- ☐ 01 Significantly worsened
- ☐ 02 Somewhat worsened
- ☐ 03 No change
- ☐ 04 Somewhat improved
- ☐ 05 Significantly improved

(*Participants completing Section C ➔ skip to END*)

© 2022 Bastain TM et al. *JAMA Network Open*

Section D. Impacts of the COVID-19 Outbreak on Pregnancy - Recall

The following questions are about your recent pregnancy.

1. Which of the following changes did you experience as a result of the COVID-19 outbreak? (***Mark all that apply***)

☐01 I changed from planning a vaginal birth to a C-section
☐02 My planned C-section or labor induction was changed
☐03 I delivered in the hospital instead of at home
☐04 I delivered at home instead of in the hospital
☐05 My healthcare provider canceled some or all of my prenatal visits
☐06 I had more prenatal visits
☐07 My prenatal visits changed from in-person to phone or telemedicine/video
☐08 My support people (e.g., spouse/partner, family) were not permitted to attend delivery or visit after delivery
☐09 I was separated from my baby immediately after delivery
☐10 I changed from planning to breastfeed to feeding only formula
☐11 I changed from planning to feed only formula to breastfeeding
☐12 Nothing changed in my prenatal care, birth or newborn plans

2. In general, how stressed were you about **changes to your birth and newborn experiences** due to the COVID-19 outbreak?

☐01 Not at all
☐02 Mildly
☐03 Moderately
☐04 Extremely

3. How did the support you received from your **prenatal care provider(s)** change due to the COVID-19 outbreak?

☐01 Significantly worsened
☐02 Somewhat worsened
☐03 No change
☐04 Somewhat improved
☐05 Significantly improved

Setting			Mode	
☐01 Clinic or site	☐02 Phone	☐03 Other location	☐01 Self-administered	☐02 Staff-administered

© 2022 Bastain TM et al. *JAMA Network Open*

eTable 2. Secondary Pandemic-Associated Traumatic Stress Outcomes in Mothers of ECHO Children by Cluster and Overall

Pandemic-Related Traumatic Stress Outcomes	Overall (N=11,473)	Low Change Cluster (N=3061)	High Change Cluster (N=8412)
**Total Sum – general severity of effects of stress**			
Mean (SD)	21.02 (6.95)	18.51 (6.98)	21.94 (6.71)
Minimum – Maximum	1 to 50	1 to 50	1 to 50
Missing	675 (5.88)	521 (6.19)	154 (5.03)
**Categorical Score – likelihood of DSM5 diagnosis of Acute Stress Disorder**			
Yes (9 or more Total Symptoms)	339 (2.95)	90 (2.94)	249 (2.96)
No (8 or less Total Symptoms)	10,459 (91.16)	2,817 (92.03)	7,642 (90.85)
Missing	675 (5.88)	154 (5.03)	521 (6.19)
**Symptom Categories – extent of effects of stress**			
Mean (SD)	2.19 (1.38)	1.67 (1.42)	2.39 (1.32)
0	1510 (13.16)	770 (25.16)	740 (8.8)
1	2,168 (18.9)	728 (23.78)	1,440 (17.12)
2	2,345 (20.44)	576 (18.82)	1,769 (21.03)
3	2,553 (22.25)	439 (14.34)	2,114 (25.13)
4	1,943 (16.94)	317 (10.36)	1,626 (19.33)
5	279 (2.43)	77 (2.52)	202 (2.4)
Missing	675 (5.88)	154 (5.03)	521 (6.19)

© 2022 Bastain TM et al. *JAMA Network Open*

eTable 3. Association Between Pandemic-related Hardships/Daily Life Changes, Coping Strategies, and Behavior Changes and Pandemic-Related Traumatic Stress Total Sum Score Among Mothers of ECHO Children[1]

	Total Sum Score (0-50)		
	**Overall**	**LoChange Cluster**	**HiChange Cluster**
Total number of mothers	N= 10675	N= 2882	N=7793
**HARDSHIPS/DAILY LIFE CHANGES**			
**COVID affected healthcare**	1.02(0.93,1.12)	1.32(1.01,1.67)	0.89(0.74,1.09)
**Greater Social Isolation**			
Quarantine from household	0.41(0.32,0.49)	0.38(0.17,0.62)	0.44(0.27,0.59)
Less contact with family	0.72(0.64,0.80)	0.18(-0.10,0.46)	Dropped
Less contact with friends	0.60(0.52,0.67)	Dropped	Dropped
Less contact with colleagues	0.69(0.59,0.77)	Dropped	Dropped
Less community events	0.53(0.45,0.61)	Dropped	Dropped
**Work related**			
Work remotely	0.31(0.21,0.40)	Dropped	-0.17(-0.32,-0.01)
Job loss or less hours	0.70(0.59,0.80)	1.16(0.91,1.49)	0.74(0.59,0.92)
New job or more hours	0.14(0.06,0.22)	Dropped	Dropped
High COVID risk	0.37(0.29,0.46)	0.40(0.16,0.66)	0.37(0.22,0.54)
Laid off employees	Dropped	Dropped	Dropped
**COVID infection**			
COVID in household	0.16(0.10,0.22)	0.41(0.22,0.61)	Dropped
Self COVID positive	Dropped	0.28(0.10,0.44)	Dropped
**COPING STRATEGIES**			
Meditation	0.50(0.41,0.58)	0.55(0.33,0.78)	0.53(0.38,0.70)
Talking with friends	0.68(0.59,0.77)	1.34(1.04,1.64)	0.19(0.05,0.34)
More family activities	Dropped	0.54(0.32,0.78)	-0.56(-0.71,-0.41)
Reading books	0.28(0.19,0.37)	0.31(0.07,0.52)	Dropped
Talking with healthcare provider	0.51(0.44,0.59)	0.57(0.40,0.76)	0.71(0.60,0.84)
Volunteering	Dropped	Dropped	Dropped
More Screentime	1.24(1.14,1.34)	1.29(1.04,1.56)	1.27(1.11,1.46)
More eating	1.20(1.11,1.30)	1.32(1.05,1.58)	1.46(1.30,1.62)
Alcohol Use	0.64(0.57,0.71)	0.43(0.29,0.60)	0.84(0.71,1.01)
Tobacco Use	0.16(0.12,0.20)	0.35(0.23,0.50)	0.20(0.14,0.27)
Marijuana Use	0.22(0.17,0.27)	0.20(0.09,0.33)	0.33(0.26,0.42)
**BEHAVIOR CHANGES**			
More home-cooked meals	0.48(0.38,0.57)	0.73(0.47,0.99)	Dropped
More takeout	0.41(0.34,0.49)	0.52(0.34,0.73)	0.41(0.27,0.55)
More physical exercise	Dropped	0.14(-0.08,0.34)	-0.38(-0.51,-0.24)
Less physical exercise	0.95(0.84,1.04)	0.76(0.47,1.01)	1.03(0.90,1.18)
More time outdoors	Dropped	0.16(-0.07,0.40)	-0.40(-0.53,-0.26)

© 2022 Bastain TM et al. *JAMA Network Open*

Less time outside	0.70(0.61,0.80)	0.72(0.46,0.98)	0.84(0.71,0.97)
No changes in behavior	-0.54(-0.60,-0.49)	-1.28(-1.49,-1.05)	Dropped

[1] Sparse partial least squares (SPLS) regression models conduct variable selection and shrinkage for variables that are determined to be relevant predictors of the outcome, relative to the other inputs in the model. This results in some variables being “dropped” from the models, noted in the above table.

© 2022 Bastain TM et al. *JAMA Network Open*

eTable 4. Association Between Pandemic-Related Stressors, Coping Strategies, and Behavior Changes and Pandemic-Related Traumatic Stress Symptoms Categories[1]

Pandemic Hardships, Coping Strategies, and Behavior Changes	Symptom Categories (0-5)		
	**Overall**	**LoChange Cluster**	**HiChange Cluster**
Total number of mothers	N= 10675	N= 2882	N=7793
**STRUCTURAL FACTORS**			
**COVID effects on healthcare:**			
COVID affected healthcare	0.18(0.17,0.20)	0.23(0.17,0.29)	0.15(0.12,0.19)
**Greater Social Isolation**			
Quarantine from household	0.06(0.05,0.08)	0.07(0.03,0.12)	0.05(0.02,0.08)
Less contact with family	0.15(0.14,0.17)	0.05(-0.00,0.11)	0.03(0.02,0.05)
Less contact with friends	0.12(0.11,0.14)	Dropped	Dropped
Less contact with colleagues	0.15(0.13,0.17)	Dropped	0.03(0.00,0.05)
Less community events	0.11(0.10,0.13)	Dropped	Dropped
**Work related**			
Work remotely	0.08(0.06,0.09)	Dropped	Dropped
Job loss or less hours	0.13(0.11,0.15)	0.23(0.18,0.31)	0.13(0.10,0.16)
New job or more hours	Dropped	Dropped	Dropped
High COVID risk	0.06(0.05,0.08)	0.08(0.03,0.13)	0.05(0.02,0.08)
Laid off employees	Dropped	Dropped	Dropped
**COVID infection**			
COVID in household	0.03(0.02,0.04)	0.08(0.04,0.12)	Dropped
Adult COVID positive	Dropped	0.05(0.02,0.09)	Dropped
**BEHAVIOR CHANGES**			
**Coping Strategies**			
Cope: Meditation	0.09(0.07,0.10)	0.10(0.06,0.15)	0.08(0.05,0.11)
Cope: Talking with friends	0.13(0.11,0.15)	0.26(0.20,0.32)	0.03(-0.00,0.05)
Cope: More family activities	Dropped	0.11(0.06,0.17)	-0.10(-0.13,-0.08)
Cope: Reading books	0.05(0.03,0.07)	0.08(0.03,0.12)	Dropped
Cope: Healthcare provider	0.08(0.07,0.09)	0.09(0.06,0.12)	0.11(0.09,0.13)
Cope: volunteering	Dropped	Dropped	Dropped
Cope: Screentime	0.23(0.21,0.25)	0.27(0.22,0.33)	0.22(0.20,0.26)
Cope: More eating	0.21(0.19,0.23)	0.24(0.20,0.30)	0.25(0.22,0.28)
Cope: Alcohol	0.11(0.10,0.13)	0.08(0.05,0.12)	0.14(0.11,0.17)
Cope: Tobacco	Dropped	0.06(0.04,0.09)	0.03(0.02,0.04)
Cope: Marijuana	0.04(0.03,0.04)	0.04(0.02,0.06)	0.05(0.04,0.06)
**Quarantine-Related Behavior Changes**			

More home-cooked meals	0.10(0.08,0.11)	0.13(0.08,0.18)	Dropped
More takeout	0.07(0.06,0.09)	0.11(0.08,0.16)	0.06(0.04,0.09)
More physical exercise	Dropped	Dropped	-0.06(-0.09,-0.03)
Less physical exercise	0.18(0.16,0.19)	0.15(0.09,0.20)	0.18(0.15,0.21)
More time outdoors	Dropped	Dropped	-0.07(-0.10,-0.04)
Less time outside	0.14(0.12,0.15)	0.15(0.10,0.21)	0.16(0.14,0.18)
No changes in behavior	-0.11(-0.12,-0.10)	-0.25(-0.29,-0.20)	Dropped

[1] Sparse partial least squares (SPLS) regression models conduct variable selection and shrinkage for variables that are determined to be relevant predictors of the outcome, relative to the other inputs in the model. This results in some variables being “dropped” from the models, noted in the above table.

© 2022 Bastain TM et al. *JAMA Network Open*

eMethods

Measures

COVID-19 Pandemic Experiences. In March-April2020, ECHO developed and released a publicly-available COVID-19 questionnaire to assess pandemic impacts on families' health, healthcare utilization, parental work and finances, health-related behaviors as well as coping strategies.[1] All COVID-19 related measures come from this instrument.

COVID-19 Pandemic-Related Traumatic Stress. The ECHO COVID-19 questionnaire includes the 10-item Pandemic-related Traumatic Stress Scale (PTSS) which is novel measure of symptoms of traumatic stress specific to the COVID-19 pandemic[2] based on the Diagnostic and Statistical Manual of Mental Disorders, 5th Edition (DSM-5)[3] criteria for Acute Stress Disorder. Acute Stress Disorder is characterized by the presence, following exposure to an event that threatens one's life or of a loved one, of symptoms representing five domains relevant for traumatic stress. To capture symptoms resembling acute stress reactions during the ongoing pandemic, items surveyed traumatic stress symptoms in five domains defined in the DSM: intrusion; negative mood; disassociation; avoidance; and difficulty regulating arousal. Responses to each item range from not at all (1) to very often (5). The specific items are as follows:

Since becoming aware of the COVID-19 outbreak, how often have you

…felt happy and satisfied with your life?

…had difficulty sleeping?

…startled easily?

…had angry outbursts?

…felt a sense of time slowing down?

…felt in a daze?

…tried to avoid thoughts and feelings about COVID-19?

…tried to avoid reading or watching information about COVID-19?

© 2022 Bastain TM et al. *JAMA Network Open*

…had distressing dreams about COVID-19?
…been distressed when I see something that reminds me of COVID-19?

Individual items were summarized as: (1) A *total sum* score (range: 5 to 50) that reflects general stress severity; (2) A *total significant symptoms* score, in which each symptom item was re-scored as "1" if participants "sometimes," "often" or "very often" reported the symptom (item score ≥3) or "0" if a symptom was "rarely" or "not at all" experienced (item score <3). These were summed to create a *total number of clinically relevant symptoms* (range: 0 to 10), reflecting a dimensional measure of traumatic stress; and (3) A *total symptom categories* score, which reflected the number of symptom categories endorsed at a clinically significant level (range 0-5), e.g., sum of symptoms domains in which at least one within the domain had a score ≥3. The *total significant symptoms* score is used as the primary outcome in the current study, because it captures the range of pandemic-related traumatic stress across the sample, whereas the symptom categories score reflects only those with clinically significant symptoms in each category. Therefore, the *total sum* score and *symptom categories* score outcomes are included in the Online Supplement as secondary outcomes (Supplemental Tables 1, 3, 4).

Pandemic-related hardships and/or daily life impacts. A total of 13 dichotomous variables were examined including the mother or household member testing positive for SARS-CoV-2 (2 items); changes to healthcare (1 item); increased social isolation (5 items: quarantining from family, reduced contact with family, friends, and colleagues, and attending fewer community events), and changes to the mother's or partner's work (5 items: job loss or reduction of hours, working remotely, getting a new job or more hours, laying off employees, or work environment placing mothers/partners at increased COVID-19 risk).

Adaptive/maladaptive coping strategies and behavior/lifestyle changes. A total of 11 coping strategies and 7 behavior/lifestyle changes—with responses of yes/no—were included. Mothers reported whether they engaged in any of the following *coping strategies* during the

pandemic (meditation, talking with friends, family activities, reading books, increased healthcare contact, volunteering, screen time, more eating, and use of alcohol, tobacco, or marijuana). They also reported on *behavior/lifestyle changes* during the COVID-19 pandemic compared to pre-pandemic (more/less home-cooking, takeout, exercise and time outdoors; or no behavior changes.

Statistical Analysis

All analyses were conducted using R statistical software 3.6.2. K-means clustering was conducted using the packages “cluster”, “fpc”, and “factoextra”. Sparse Partial Least Squares regression was conducted using the package “spls”.

K-means clustering

In this cross-sectional study, we characterized clusters of mothers based on their responses to 31 pandemic hardships, coping strategies, and lifestyle/behavior changes using k-means clustering,[4,5]an unsupervised machine learning method that groups individuals based on similarities in patterns of responses to specified inputs. In this case, the 31 inputs are responses to the COVID-19 questionnaires (pandemic-related hardships, coping strategies, and behavior changes), independent of any outcome of interest. The optimal number of clusters was selected based on scores from a Silhouette analysis,[6,7] which estimates the similarity of data points within clusters versus between clusters; the number of clusters that maximizes the silhouette analysis score is the optimal number of clusters.

We then described the sociodemographic characteristics of these clusters to determine if there were fundamental differences in characteristics by cluster with respect to maternal race, ethnicity, marital status, educational attainment, and/or household income.

© 2022 Bastain TM et al. *JAMA Network Open*

SPLS Regression

Finally, we conducted sparse partial least squares (SPLS) regression[41] [8]analysis of the relationship between responses to COVID-19 pandemic related hardships, coping strategies and behavior changes with reported pandemic-related traumatic stress, adjusting for cohort recruitment site and state of residence to account for unique cohort related characteristics and geographic variation in COVID-19 pandemic impacts, respectively. Mothers were excluded from the SPLS analysis if they were missing the outcome of pandemic-related traumatic stress (n=675) or missing state of residence (n=123).

SPLS is a supervised machine learning approach that 1) allows inclusion of all potential variables into a single regression model and 2) conducts variable selection and shrinkage based on whether or not inputs are determined to be associated with the outcome, relative to the other inputs in the model. The optimal number of variable groupings and penalty for primary analyses was determined with K-fold cross validation, determined based on the values that minimize mean squared prediction error of the model. This method results in the exclusion of some variables from the final summary tables (noted as "dropped" in regression tables). Bootstrapping was used to calculate 95% confidence intervals. We further determined whether the association between responses to pandemic-related hardships and pandemic-related traumatic stress differed by cluster membership by running stratified, cluster-specific SPLS models. Clusters are fully independent of one another in that an individual cannot be a member of both clusters. Therefore, we used the approach described by Altman.[9] Briefly, the difference between clusters is calculated as the absolute value of the difference in the coefficients, or abs(b1 – b2), where b1 is the estimated coefficient for a hardship in cluster 1 and b2 is the estimates coefficient for that same hardship in cluster 2. The standard error is approximated as the square root of the sum of squared standard errors from each cluster, or SE = sqrt(SE1^2 + SE2^2). Because PLS provides bootstrapped confidence intervals, we calculated the SE1 and SE2 from the bounds of

© 2022 Bastain TM et al. *JAMA Network Open*

the 95% confidence intervals. The estimates SE was the average of the SE obtained from the upper and lower bounds of the bootstrapped 95% confidence intervals.

RESULTS

eTable 2 describes the distribution of secondary outcomes by cluster and overall. Mean total sum of PTS is 19.78 (SD 8.36), with a mean of 17.58 (SD 7.91) in the LoChange Cluster and 20.58 (SD 8.38) in the HiChange cluster. Mean symptoms categories score was 2.06 (1.44) overall, 1.58 (1.43) in the LoChange cluster, and 2.24 (1.40) in the HiChange cluster.

© 2022 Bastain TM et al. *JAMA Network Open*

eFigure 2. Optimal Number of Clusters by Total Within Sum of Squares (Panel A) and Silhouette Width (Panel B)

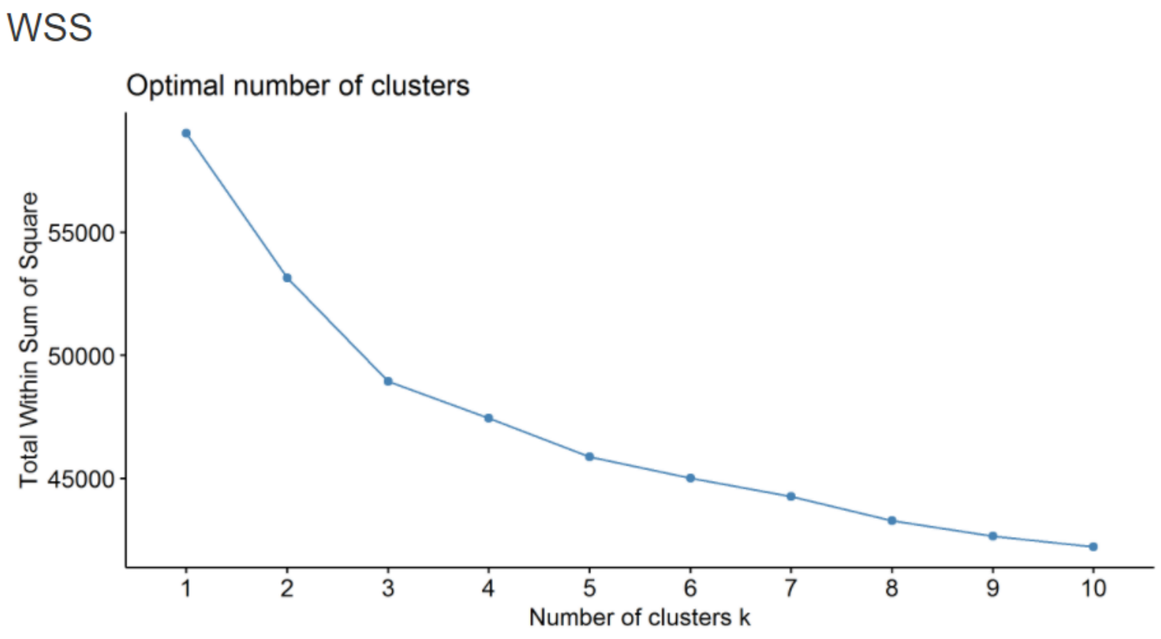

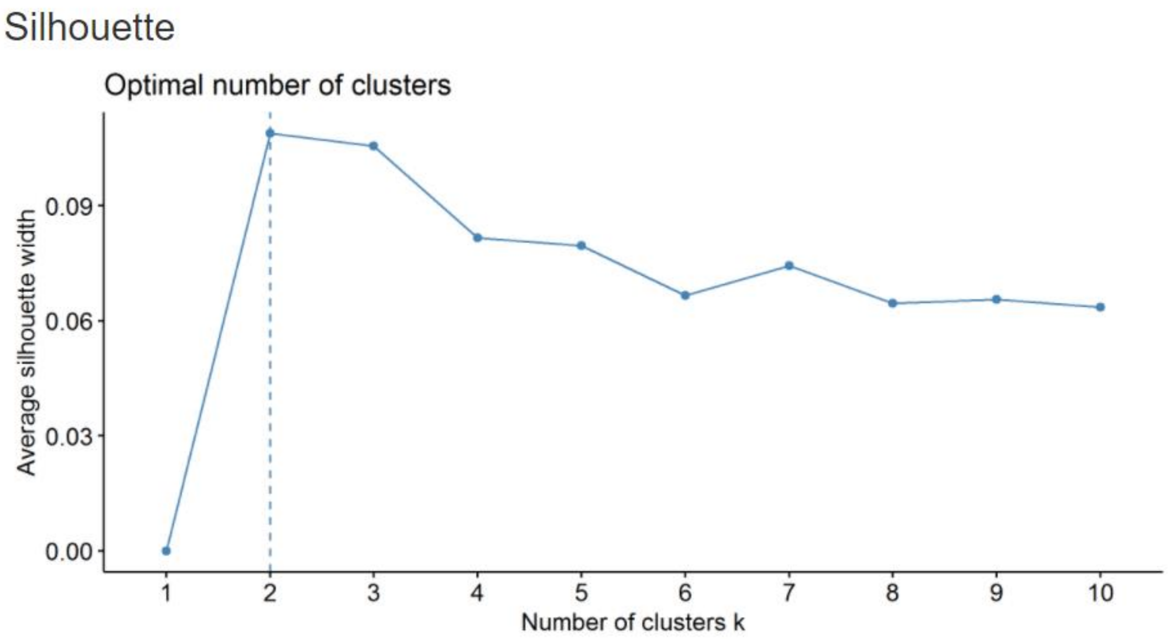

© 2022 Bastain TM et al. *JAMA Network Open*

eTable 5. Pandemic-Related Hardships, Coping Strategies and Behavior Changes By Cluster and Overall Reported by ECHO Mothers

Pandemic Hardships, Coping Strategies, and Behavior Changes	Overall (N=11,473) N (%)	LoChange Cluster (N=3061) N (%)	HiChange Cluster (N=8412) N (%)
**HARDSHIPS/DAILY LIFE CHANGES**			
**COVID Affected Healthcare**	6,693 (58.34)	1,045 (34.14)	5,648 (67.14)
**Greater Social Isolation**			
Quarantine from household	2,205 (19.22)	332 (10.85)	1,873 (22.27)
Family	8,728 (76.07)	993 (32.44)	7,735 (91.95)
Friends	9,368 (81.65)	1,165 (38.06)	8,203 (97.52)
Work colleagues	6,823 (59.47)	333 (10.88)	6,490 (77.15)
Community events	9,195 (80.14)	1,257 (41.07)	7,938 (94.37)
**Work related**			
Work remotely	5,190 (45.24)	504 (16.47)	4,686 (55.71)
Job loss or reduction	4,531 (39.49)	942 (30.77)	3,589 (42.67)
New job or more hours	2,321 (20.23)	474 (15.49)	1,847 (21.96)
High COVID risk	2,967 (25.86)	542 (17.71)	2,425 (28.83)
Laid off employees	431 (3.76)	86 (2.81)	345 (4.1)
**COVID infection**			
COVID+ in household	1,147 (10)	277 (9.05)	870 (10.34)
Self COVID+	861 (7.5)	221 (7.22)	640 (7.61)
**COPING STRATEGIES**			
Meditation	2,592 (22.59)	395 (12.9)	2,197 (26.12)
Friend/family communication	8,088 (70.5)	1,375 (44.92)	6,713 (79.8)
Family activities	4,748 (41.38)	694 (22.67)	4,054 (48.19)
Reading/puzzles	3,698 (32.23)	485 (15.84)	3,213 (38.2)
Increased healthcare contact	993 (8.66)	146 (4.77)	847 (10.07)
Volunteering	428 (3.73)	45 (1.47)	383 (4.55)
More screentime	5,464 (47.62)	660 (21.56)	4,804 (57.11)
More eating	3,801 (33.13)	494 (16.14)	3,307 (39.31)
Alcohol use	1,769 (15.42)	152 (4.97)	1,617 (19.22)
Tobacco use	337 (2.94)	97 (3.17)	240 (2.85)
Marijuana use	422 (3.68)	73 (2.38)	349 (4.15)
**BEHAVIOR CHANGES**			
More home cooking	7,916 (69)	1,306 (42.67)	6,610 (78.58)
More takeout	2,423 (21.12)	396 (12.94)	2,027 (24.1)
More exercise	3,015 (26.28)	429 (14.02)	2,586 (30.74)
Less exercise	4,457 (38.85)	558 (18.23)	3,899 (46.35)
More time outdoors	4,712 (41.07)	690 (22.54)	4,022 (47.81)
Less time outdoors	3,476 (30.3)	617 (20.16)	2,859 (33.99)
No change	1,139 (9.93)	957 (31.26)	182 (2.16)

© 2022 Bastain TM et al. *JAMA Network Open*

eFigure 3. Number of Total Significant Symptoms of Pandemic-Related Traumatic Stress by Cluster in Mothers of ECHO Children

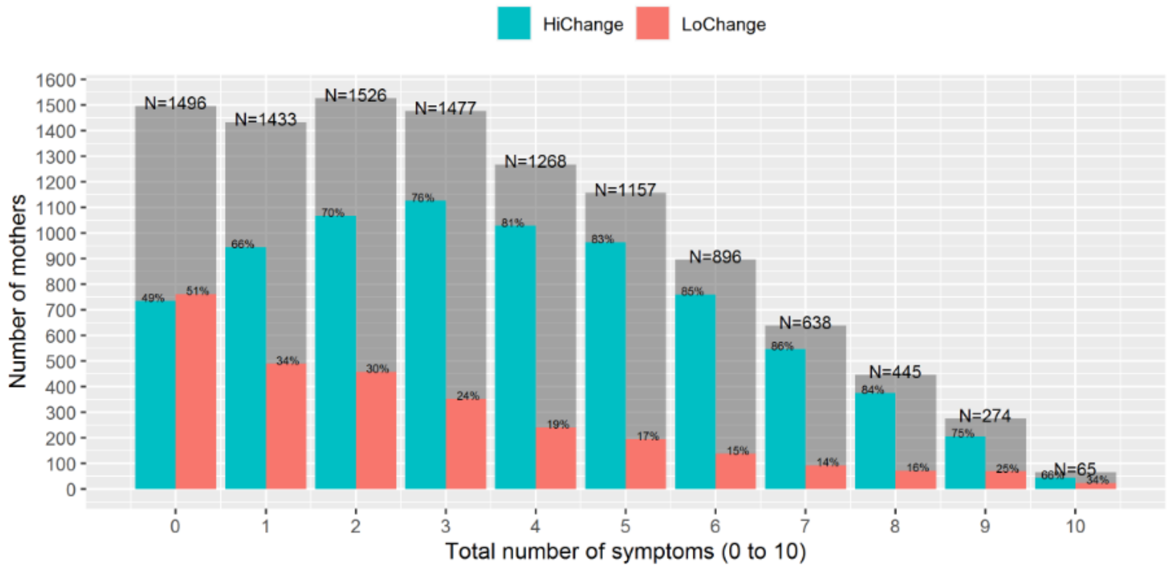

© 2022 Bastain TM et al. *JAMA Network Open*

eReferences

1. Environmental influences on Child Health Outcomes (ECHO) Program. PhenX Toolkit ECHO COVID-19 Questionnaires. 2020; https://www.phenxtoolkit.org/covid19/source. Accessed February 26, 2022.
2. Margolis A, Algermissen M, Herbstman J, et al. Acute Stress Questions for ECHO COVID-19 Survey. *PsychArchives.* 2021;https://doi.org/10.23668/PSYCHARCHIVES.5100.
3. American Psychiatric Association. Diagnostic and statistical manual of mental disorders (5th ed.). *https://doiorg/101176/appibooks9780890425596.* 2013.
4. Forgy EW. Cluster Analysis of Multivariate Data - Efficiency Vs Interpretability of Classifications. *Biometrics.* 1965;21(3):768-&.
5. Maechler M, Rousseeuw P, Struyf A, Hubert M, K H. cluster: Cluster Analysis Basics and Extensions. R package version 2.1.2 — For new features, see the 'Changelog' file (in the package source), https://CRAN.R-project.org/package=cluster. 2021.
6. de Amorim RC, Hennig C. Recovering the number of clusters in data sets with noise features using feature rescaling factors. *Information Sciences.* 2015;324:126-145.
7. Rousseeuw PJ. Silhouettes: A graphical aid to the interpretation and validation of cluster analysis. *Journal of Computational and Applied Mathematics.* 1987;20:53-65.
8. Chun H, Keleş S. Sparse partial least squares regression for simultaneous dimension reduction and variable selection. *J R Stat Soc Series B Stat Methodol.* 2010;72(1):3-25.
9. Altman DG, Bland JM. Interaction revisited: the difference between two estimates. *BMJ.* 2003;326(7382):219.

© 2022 Bastain TM et al. *JAMA Network Open*
