# Supplementary material for: COVID-19 Pandemic Experiences and Symptoms of Pandemic-Associated Traumatic Stress Among Mothers in the US
Source: JAMA Netw Open. 2022 Dec 16;5(12):e2247330. doi: 10.1001/jamanetworkopen.2022.47330 (PMC9856510; doi:10.1001/jamanetworkopen.2022.47330)
Supplement: Supplement 2. — Nonauthor Collaborators [file jamanetwopen-e2247330-s002.pdf]

Supplemental Online Content: Nonauthor Collaborators
*First name, last name, and suffix (if applicable) are required and will appear in PubMed.

*Group Name(s): Program Collaborators for Environmental influences on Child Health Outcomes							
***First Name and Middle Initial(s)**	***Last Name**	***Suffix (eg, Jr, III)**	Academic Degrees	Institution	Location (city, state/province, country)	Role or Contribution, eg, chair, principal investigator	Group (if more than 1 Group listed in the byline) and/or Subgroup (eg, Steering Committee)
Brian P	Smith		MD	Duke Clinical Research Institute	Durham, North Carolina, USA	ECHO Coordinating Center Prinicipal Investigator	ECHO Coordinating Center U2COD023375
Kristen L	Newby		MD	Duke Clinical Research Institute	Durham, North Carolina, USA	ECHO Coordinating Center Prinicipal Investigator	ECHO Coordinating Center U2COD023375
Lisa P	Jacobson		PhD	Johns Hopkins University	Baltimore, Maryland, USA	ECHO Data Analysis Center Principal Investigator	ECHO Data Analysis Center U24D023382
Corette B	Parker		PhD	Research Triangle Park Institute	Durham, North Carolina, USA	ECHO Data Analysis Center Principal Investigator	ECHO Data Analysis Center U24D023382
Richard C	Gershon		PhD	Northwestern University School of Medicine	Evanston, Illinois, USA	ECHO Person Reported Outcome Core Principal Investigator	ECHO Person Reported Outcome Core U24OD023319
David	Cella		PhD	Northwestern University School of Medicine	Evanston, Illinois, USA	ECHO Person Reported Outcome Core Principal Investigator	ECHO Person Reported Outcome Core U24OD023319
Akram N	Alshawabkeh		PhD	Northeastern University	Boston, Massachusetts, USA	ECHO Cohort Principal Investigator	ECHO Cohort UH3OD023251
Judy L	Aschner		MD	Albert Einstein College of Medicine	Bronx, New York, USA	ECHO Cohort Principal Investigator	ECHO Cohort UH3OD023320
Stephanie L	Merhar		MD	Cincinnati Children's Hospital Medical Center	Cincinnati, Ohio, USA	ECHO Cohort Principal Investigator	ECHO Cohort UH3OD023320
Clement L	Ren		MD	Indiana University, Riley Hospital for Children	Indianapolis, IN, USA	ECHO Cohort Principal Investigator	ECHO Cohort UH3OD023320

Supplemental Online Content: Nonauthor Collaborators
*First name, last name, and suffix (if applicable) are required and will appear in PubMed.

*First Name and Middle Initial(s)	*Last Name	*Suffix (eg, Jr, III)	Academic Degrees	Institution	Location (city, state/province, country)	Role or Contribution, eg, chair, principal investigator	Group (if more than 1 Group listed in the byline) and/or Subgroup (eg, Steering Committee)
Anne Marie	Reynolds		MD	University of Buffalo, Jacobson School of Medicine and Biomedical Sciences	Buffalo, NY, USA	ECHO Cohort Principal Investigator	ECHO Cohort UH3OD023320
Roberta	Keller		MD	University of California	San Francisco, CA, USA	ECHO Cohort Principal Investigator	ECHO Cohort UH3OD023320
Gloria S	Pryhuber		MD	University of Rochester Medical Center	Rochester, NY, USA	ECHO Cohort Principal Investigator	ECHO Cohort UH3OD023320
Andrea J	Duncan		MD	University of Texas Health Sciences Center	Houston, TX, USA	ECHO Cohort Principal Investigator	ECHO Cohort UH3OD023320
Paul E	Moore		MD	Vanderbilt Children’s Hospital	Nashville, TN, USA	ECHO Cohort Principal Investigator	ECHO Cohort UH3OD023320
Andrea L	Lampland		MD	Children’s Hospital and Clinic	Minneapolis, MN, USA	ECHO Cohort Principal Investigator	ECHO Cohort UH3OD023320
Rajan	Wadhawan		MD	Florida Hospital for Children	Orlando, FL, USA	ECHO Cohort Principal Investigator	ECHO Cohort UH3OD023320
Carol L	Wagner		MD	Medical University of South Carolina	Charleston, SC, USA	ECHO Cohort Principal Investigator	ECHO Cohort UH3OD023320
Mark L	Hudak		MD	University of Florida College of Medicine	Jacksonville, FL, USA	ECHO Cohort Principal Investigator	ECHO Cohort UH3OD023320
Dennis E	Mayock		MD	University of Washington	Seattle, WA, USA	ECHO Cohort Principal Investigator	ECHO Cohort UH3OD023320
Lisa K	Walshburn		MD	Wake Forest University School of Medicine	Winston Salem, NC	ECHO Cohort Principal Investigator	ECHO Cohort UH3OD023320
Susan L	Teitelbaum		PhD	Icahn School of Medicine at Mount Sinai	New York, NY, USA	ECHO Cohort Principal Investigator	ECHO Cohort UH3OD023320
Annemarie	Stroustrup		MD	Icahn School of Medicine at Mount Sinai	New York, NY, USA	ECHO Cohort Principal Investigator	ECHO Cohort UH3OD023320
Clancy B	Blair		PhD	New York University	New York, NY, USA	ECHO Cohort Principal Investigator	ECHO Cohort UH3OD023332
Lisa	Gatzke-Kopp		PhD	Pennsylvania State University	University Park, PA, USA	ECHO Cohort Principal Investigator	ECHO Cohort UH3OD023332

Supplemental Online Content: Nonauthor Collaborators
*First name, last name, and suffix (if applicable) are required and will appear in PubMed.

***First Name and Middle Initial(s)**	***Last Name**	***Suffix (eg, Jr, III)**	Academic Degrees	Institution	Location (city, state/province, country)	Role or Contribution, eg, chair, principal investigator	Group (if more than 1 Group listed in the byline) and/or Subgroup (eg, Steering Committee)
Margaret M	Swingler		PhD	University of North Carolina	Chapel Hill, NC, USA	ECHO Cohort Principal Investigator	ECHO Cohort UH3OD023332
Carlos	Camargo		MD	Massachusetts General Hospital	Boston, MA, USA	ECHO Cohort Principal Investigator	ECHO Cohort UH3OD023253
Jonathan M	Mansbach		MD	Boston Children's Hospital	Boston, MA, USA	ECHO Cohort Principal Investigator	ECHO Cohort UH3OD023253
Jonathan M	Spergel		MD	Children's Hospital of Philadelphia	Philadelphia, PA, USA	ECHO Cohort Principal Investigator	ECHO Cohort UH3OD023253
Michelle D	Stevenson		MD	Norton Children's Hospital	Louisville, KY, USA	ECHO Cohort Principal Investigator	ECHO Cohort UH3OD023253
Cindy S	Bauer		MD	Phoenix Children's Hospital	Phoenix AZ, USA	ECHO Cohort Principal Investigator	ECHO Cohort UH3OD023253
Dana	Dabelea		MD	University of Colorado Denver	Denver, CO, USA	ECHO Cohort Principal Investigator	ECHO Cohort UH3OD023248
Sean CL	Deoni		PhD	Memorial Hospital of Rhode Island	Providence RI, USA	ECHO Cohort Principal Investigator	ECHO Cohort UH3OD023313
Cristiane	Duarte		PhD	New York State Psychiatric Institute	New York, NY, USA	ECHO Cohort Principal Investigator	ECHO Cohort UH3OD023328
Glorisa J	Canino		PhD	University of Puerto Rico	San Jaun, PR,	ECHO Cohort Principal Investigator	ECHO Cohort UH3OD023328
Anne L	Dunlop		MD	Emory University	Atlanta, GA, USA	ECHO Cohort Principal Investigator	ECHO Cohort UH3OD023318
Amy J	Elliott		PhD	Avera Health Rapid City	Rapid City, SD, USA	ECHO Cohort Principal Investigator	ECHO Cohort UH3OD023279
Assiamira M	Ferrara		MD	Kaiser Permanente Northern California Division of Research	Oakland, CA, USA	ECHO Cohort Principal Investigator	ECHO Cohort UH3OD023289
Lisa A	Croen		PhD	Kaiser Permanente Northern California Division of Research	Oakland, CA, USA	ECHO Cohort Principal Investigator	ECHO Cohort UH3OD023289
James E	Gern		MD	University of Wisconsin	Madison WI, USA	ECHO Cohort Principal Investigator	ECHO Cohort UH3OD023282
Edward M	Zoratti		MD	Henry Ford Health System	Detroit, MI	ECHO Cohort Principal Investigator	ECHO Cohort UH3OD023282

Supplemental Online Content: Nonauthor Collaborators
*First name, last name, and suffix (if applicable) are required and will appear in PubMed.

***First Name and Middle Initial(s)**	***Last Name**	***Suffix (eg, Jr, III)**	Academic Degrees	Institution	Location (city, state/province, country)	Role or Contribution, eg, chair, principal investigator	Group (if more than 1 Group listed in the byline) and/or Subgroup (eg, Steering Committee)
Christine M	Seroogy		MD	Marshfield Clinic Research Institute	Marshfield, WI, USA	ECHO Cohort Principal Investigator	ECHO Cohort UH3OD023282
Casper G	Bendixsen		PhD	Marshfield Clinic Research Institute	Marshfield, WI, USA	ECHO Cohort Principal Investigator	ECHO Cohort UH3OD023282
Leonard B	Bacharier		MD	Boston Medical Center	Boston MA, USA	ECHO Cohort Principal Investigator	ECHO Cohort UH3OD023282
George T	O’Connor		MD	Boston Medical Center	Boston MA, USA	ECHO Cohort Principal Investigator	ECHO Cohort UH3OD023282
Meyer	Kattan		MD	Children’s Hospital of New York	New York, NY, USA	ECHO Cohort Principal Investigator	ECHO Cohort UH3OD023282
Robert A	Wood		MD	Johns Hopkins University, School of Medicine	Baltimore, MD, USA	ECHO Cohort Principal Investigator	ECHO Cohort UH3OD023282
Katherine	Rivera-Spoljaric		MD	Washington University in St Louis	St Louis, MO, USA	ECHO Cohort Principal Investigator	ECHO Cohort UH3OD023282
Tina V	Hartert		MD	Vanderbilt University	Nashville TN, USA	ECHO Cohort Principal Investigator	ECHO Cohort UH3OD023282
Christine C	Johnson		PhD	Henry Ford Health System	Detroit, MI, USA	ECHO Cohort Principal Investigator	ECHO Cohort UH3OD023282
Anne M	Singh		MD	University of Wisconsin	Madison, WI, USA	ECHO Cohort Principal Investigator	ECHO Cohort UH3OD023282
Irva	Hertz-Picciotto		MD	University of California Davis Mind Institute	Sacramento, CA, USA	ECHO Cohort Principal Investigator	ECHO Cohort UH3OD023365
Alison E	Hipwell		PhD	University of Pittsburgh	Pittsburgh, PA, USA	ECHO Cohort Principal Investigator	ECHO Cohort UH3OD023244
Caherine J	Karr		MD	University of Washington, Department of Environmental and Occupational Health Sciences	Seattle, WA, USA	ECHO Cohort Principal Investigator	ECHO Cohort UH3OD023271
Alex	Mason		PhD	University of Tennessee Health Science Center	Memphis, TN, USA	ECHO Cohort Principal Investigator	ECHO Cohort UH3OD023271
Sheela	Sathyanarayana		MD	Seattle Children’s Research Institute	Seattle, WA	ECHO Cohort Principal Investigator	ECHO Cohort UH3OD023271

Supplemental Online Content: Nonauthor Collaborators
*First name, last name, and suffix (if applicable) are required and will appear in PubMed.

*First Name and Middle Initial(s)	*Last Name	*Suffix (eg, Jr, III)	Academic Degrees	Institution	Location (city, state/province, country)	Role or Contribution, eg, chair, principal investigator	Group (if more than 1 Group listed in the byline) and/or Subgroup (eg, Steering Committee)
Barry M	Lester		PhD	Women & Infants Hospital of Rhode Island	Providence RI, USA	ECHO Cohort Principal Investigator	ECHO Cohort UH3OD023347
Brian S	Carter		MD	Children’s Mercy	Kansas City, MO, USA	ECHO Cohort Principal Investigator	ECHO Cohort UH3OD023347
Carmen J	Marsit		PhD	Emory University	Atlanta, GA, USA	ECHO Cohort Principal Investigator	ECHO Cohort UH3OD023347
Steven L	Pastyrnak		PhD	Helen DeVos Children’s Hospital	Grand Rapids, MI, USA	ECHO Cohort Principal Investigator	ECHO Cohort UH3OD023347
Charles	Neal		MD	Kapiolani Medical Center for Women and Children	Providence, RI, USA	ECHO Cohort Principal Investigator	ECHO Cohort UH3OD023347
Lynne M	Smith		MD	Los Angeles Biomedical Research Institute at Harbour-UCLA Medical Center	Los Angeles CA, USA	ECHO Cohort Principal Investigator	ECHO Cohort UH3OD023347
Jennifer B	Helderman		MD	Wake Forest University School of Medicine	Winston Salem, NC	ECHO Cohort Principal Investigator	ECHO Cohort UH3OD023347
Leslie D	Leve		PhD	Prevention Science Institute, University of Oregon	Eugene, OR, USA	ECHO Cohort Principal Investigator	ECHO Cohort UH3OD023389
Jenae M	Neiderhiser		PhD	Pennsylvania State University	University Park, PA, USA	ECHO Cohort Principal Investigator	ECHO Cohort UH3OD023389
Scott T	Weiss		MD	Brigham and Women's Hospital	Boston, MA, USA	ECHO Cohort Principal Investigator	ECHO Cohort UH3OD023268
Robert	Zeiger		MD	Kaiser Permanente, Southern California	San Diego, CA, USA	ECHO Cohort Principal Investigator	ECHO Cohort UH3OD023268
Cindy	McEvoy		MD	Oregon Health and Science University	Portland, OR, USA	ECHO Cohort Principal Investigator	ECHO Cohort UH3OD023288
Robert S	Tepper		MD	Indiana University, Riley Hospital for Children	Indianapolis, IN, USA	ECHO Cohort Principal Investigator	ECHO Cohort UH3OD023288
Kristen	Lyall		ScD	Drexel Autism Institute	Philadelphia, PA, USA	ECHO Cohort Principal Investigator	ECHO Cohort UH3OD023342
Rebecca	Landa		PhD	Johns Hopkins Bloomberg School of Public Health Kennedy Krieger Institute	Baltimore, MD, USA	ECHO Cohort Principal Investigator	ECHO Cohort UH3OD023342

Supplemental Online Content: Nonauthor Collaborators
*First name, last name, and suffix (if applicable) are required and will appear in PubMed.

***First Name and Middle Initial(s)**	***Last Name**	***Suffix (eg, Jr, III)**	Academic Degrees	Institution	Location (city, state/province, country)	Role or Contribution, eg, chair, principal investigator	Group (if more than 1 Group listed in the byline) and/or Subgroup (eg, Steering Committee)
Sally J	Ozonoff		PhD	University of California, UC Davis Medical Center Mind Institute	Sacramento, CA, USA	ECHO Cohort Principal Investigator	ECHO Cohort UH3OD023342
Stephen R	Dager		MD	University of Washington	Seattle, WA, USA	ECHO Cohort Principal Investigator	ECHO Cohort UH3OD023342
Robert T	Schultz		PhD	Children's Hospital of Philadelphia - Center for Autism Research	Philadelphia, PA, USA	ECHO Cohort Principal Investigator	ECHO Cohort UH3OD023342
Joseph	Piven		MD	University of North Carolina at Chapel Hill	Chapel Hill, NC, USA	ECHO Cohort Principal Investigator	ECHO Cohort UH3OD023342
Heather	Volk		PhD	Johns Hopkins Bloomberg School of Public Health	Baltimore, Maryland, USA	ECHO Cohort Principal Investigator	ECHO Cohort UH3OD023342
Thomas G	O'Connor		PhD	University of Rochester Medical Center Rochester	New York, NY, USA	ECHO Cohort Principal Investigator	ECHO Cohort UH3OD023349
Hyagriv	Simhan		MD	University of Pittsburgh Medical Center, Magee Women's Hospital	Pittsburgh, PA, USA	ECHO Cohort Principal Investigator	ECHO Cohort UH3OD023349
Emily	Oken		MD	Harvard Pilgrim Health Care Institute	Boston, MA, USA	ECHO Cohort Principal Investigator	ECHO Cohort UH3OD023286
Michael	O'Shea		MD	University of North Carolina	Chapel Hill, NC	ECHO Cohort Principal Investigator	ECHO Cohort UH3OD023348
Ruben	Vaidya		MD	Baystate Children's Hospital	Springfield, MA, USA	ECHO Cohort Principal Investigator	ECHO Cohort UH3OD023348
Rawad	Obeid		MD	Beaumont Health Medical Center	Royal Oak, MI, USA	ECHO Cohort Principal Investigator	ECHO Cohort UH3OD023348
Caitlin	Rollins		MD	Boston Children's Hospital	Boston, MA, USA	ECHO Cohort Principal Investigator	ECHO Cohort UH3OD023348
Kelly A	Bear		DO	East Carolina University Brody School of Medicine	Greenville, NC	ECHO Cohort Principal Investigator	ECHO Cohort UH3OD023348
Madeleine	Lenski		MS	Michigan State University College of Human Medicine	East Lansing, MI, USA	ECHO Cohort Principal Investigator	ECHO Cohort UH3OD023348
Michael E	Msall		MD	University of Chicago	Chicago IL, USA	ECHO Cohort Principal Investigator	ECHO Cohort UH3OD023348
Lisa K	Washburn		MD	Wake Forest Baptist Health (Atrium Health),	Winston Salem, NC	ECHO Cohort Principal Investigator	ECHO Cohort UH3OD023348

*First name, last name, and suffix (if applicable) are required and will appear in PubMed.

***First Name and Middle Initial(s)**	***Last Name**	***Suffix (eg, Jr, III)**	Academic Degrees	Institution	Location (city, state/province, country)	Role or Contribution, eg, chair, principal investigator	Group (if more than 1 Group listed in the byline) and/or Subgroup (eg, Steering Committee)
Angela M	Montgomery		MD	Yale School of Medicine	New Haven, CT, USA	ECHO Cohort Principal Investigator	ECHO Cohort UH3OD023348
Jean	Kerver		PhD	Michigan State University	East Lansing, MI, USA	ECHO Cohort Principal Investigator	ECHO Cohort UH3OD023285
Charles	Barone		MD	Henry Ford Health System	Detroit, MI, USA	ECHO Cohort Principal Investigator	ECHO Cohort UH3OD023285
Patricia	McKane		DVM	Michigan Department of Health and Human Services	Lansing, MI, USA	ECHO Cohort Principal Investigator	ECHO Cohort UH3OD023285
Nigel	Paneth		MD	Michigan State University	East Lansing, MI, USA	ECHO Cohort Principal Investigator	ECHO Cohort UH3OD023285
Michael R	Elliott		PhD	University of Michigan	Ann Arbor, MI, USA	ECHO Cohort Principal Investigator	ECHO Cohort UH3OD023285
Susan L	Schantz		PhD	University of Illinois, Beckman Institute	Urbana, IL, USA	ECHO Cohort Principal Investigator	ECHO Cohort UH3OD023272
Tracey J	Woodruff		PhD	University of California, San Francisco	San Francisco, CA, USA	ECHO Cohort Principal Investigator	ECHO Cohort UH3OD023272
Joseph B	Stanford		MD	University of Utah	Salt Lake City, UT, USA	ECHO Cohort Principal Investigator	ECHO Cohort UH3OD023249
Christy A	Porucznik		PhD	University of Utah	Salt Lake City, UT, USA	ECHO Cohort Principal Investigator	ECHO Cohort UH3OD023249
Robert M	Silver		MD	University of Utah	Salt Lake City, UT, USA	ECHO Cohort Principal Investigator	ECHO Cohort UH3OD023249
Elisabeth	Conradt		PhD	University of Utah	Salt Lake City, UT, USA	ECHO Cohort Principal Investigator	ECHO Cohort UH3OD023249
Leonardo	Trasande		MD	New York School of Medicine	New York, NY, USA	ECHO Cohort Principal Investigator	ECHO Cohort UH3OD023305
Rosalind J	Wright		MD	Icahn School of Medicine at Mount Sinai	New York, NY, USA	ECHO Cohort Principal Investigator	ECHO Cohort UH3OD023337
Michelle	Bosquet-Enlow		phD	Boston Children’s Hospital	Boston MA, USA	ECHO Cohort Principal Investigator	ECHO Cohort UH3OD023337
Kathi	Huddleston		PhD	George Mason University	Fairfax, VA, USA	ECHO Cohort Principal Investigator	ECHO Cohort UH3OD023337

Supplemental Online Content: Nonauthor Collaborators

*First name, last name, and suffix (if applicable) are required and will appear in PubMed.

***First Name and Middle Initial(s)**	***Last Name**	***Suffix (eg, Jr, III)**	Academic Degrees	Institution	Location (city, state/province, country)	Role or Contribution, eg, chair, principal investigator	Group (if more than 1 Group listed in the byline) and/or Subgroup (eg, Steering Committee)
Nicole	Bush		PhD	University of California, San Francisco	San Francisco CA, USA	ECHO Cohort Principal Investigator	ECHO Cohort UH3OD023271 and UH3OD023282
Ruby HN	Nguyen		PhD	University of Minnesota	Minneapolis, MN, USA	ECHO Cohort Principal Investigator	ECHO Cohort UH3OD023271 and UH3OD023282
Emily S	Barrett		PhD	University of Rochester Medical Center	Rochester, NY, USA	ECHO Cohort Principal Investigator	ECHO Cohort UH3OD023271 and UH3OD023282
