# Supplementary material for: COVID-19 Pandemic Experiences and Symptoms of Pandemic-Associated Traumatic Stress Among Mothers in the US
Source: JAMA Netw Open. 2022 Dec 16;5(12):e2247330. doi: 10.1001/jamanetworkopen.2022.47330 (PMC9856510; doi:10.1001/jamanetworkopen.2022.47330)
Supplement: Supplement 3. — Data Sharing Statement [file jamanetwopen-e2247330-s003.pdf]

Data Sharing Statement

Bastain. COVID-19 Pandemic Experiences and Symptoms of Pandemic-Associated Traumatic Stress Among Mothers in the US. *JAMA Netw Open*. Published December 16, 2022. doi:10.1001/jamanetworkopen.2022.47330

Data
Data available: Yes
Data types: Deidentified participant data
How to access data: De-identified data from the ECHO Program are available through NICHD's Data and Specimen Hub (DASH). DASH is a centralized resource that allows researchers to access data from various studies via a controlled-access mechanism. Researchers can now request access to these data by creating a DASH account and submitting a Data Request Form. The NICHD DASH Data Access Committee will review the request and provide a response in approximately two to three weeks. Once granted access, researchers will be able to use the data for three years. See the DASH Tutorial for more detailed information on the process. https://dash.nichd.nih.gov/study/417122
When available: With publication

Supporting Documents
Document types: None

Additional Information
Who can access the data: Researchers whose proposed use of the data has been approved.
Types of analyses: The Data and Specimen Hub (DASH) system owned by the Eunice Kennedy Shriver National Institute of Child Health and Human Development is offered as an information and data resource for scientific research. Users of DASH agree to comply with all terms and conditions of the NICHD DASH User Agreement during the registration process. By accepting the NICHD DASH User Agreement, you agree: to use NICHD DASH for the purposes of archiving and accessing data obtained from scientific research with the intent of data reuse to use NICHD DASH data for scientific research in an institution with an approved assurance from the Department of Health and Human Services Office for Human Research Protections, and to not use the data for commercial purposes (or sell the data obtained from NICHD DASH) to preserve and protect the confidentiality of, and not attempt to identify, any individuals or households in the data that archived data in NICHD DASH are provided without warranty or liability of any kind to notify the NICHD DASH Administrator of any errors discovered in the archived data to establish safeguards to prevent unauthorized viewing or release of NICHD DASH information or data to comply with any charges that may apply for various services offered by NICHD DASH to ensure that the means of access to NICHD DASH (such as passwords) are kept secure and not disclosed to anyone else that personal data submitted by you are accurate to the best of your knowledge and kept up to date by you that personal data provided by you may be used for administrative management of NICHD DASH and for reporting purposes with the goal of improving services offered by NICHD DASH that any breach of the NICHD DASH User Agreement could lead to termination of your access to the services
Mechanisms of data availability: Without investigator support
